# Supplementary material for: Review of Crop Wild Relative Conservation and Use in West Asia and North Africa
Source: Plants (Basel). 2024 May 13;13(10):1343. doi: 10.3390/plants13101343 (PMC11124793; doi:10.3390/plants13101343)
Supplement: Supplementary file 1 [file plants-13-01343-s001.zip › plants-2898175-supplementary.pdf]

Supplementary Table S1. Priority 488 CWR found in the WANA region.

Family	Crop	Crop Latin Name	CWR	Genepool
Poaceae	Wheat	*Triticum aestivum* L.	*Aegilops bicornis* (Forssk.) Jaub. et Spach	Secondary
Poaceae	Wheat	*Triticum aestivum* L.	*Aegilops bicornis* (Forssk.) Jaub. et Spach var. *anathera* Eig	Secondary
Poaceae	Wheat	*Triticum aestivum* L.	*Aegilops bicornis* (Forssk.) Jaub. et Spach var. *bicornis*	Secondary
Poaceae	Wheat	*Triticum aestivum* L.	*Aegilops biuncialis* Vis.	Secondary
Poaceae	Wheat	*Triticum aestivum* L.	*Aegilops columnaris* Zhuk.	Secondary
Poaceae	Wheat	*Triticum aestivum* L.	*Aegilops comosa* Sm.	Secondary
Poaceae	Wheat	*Triticum aestivum* L.	*Aegilops crassa* Boiss.	Secondary
Poaceae	Wheat	*Triticum aestivum* L.	*Aegilops cylindrica* Host	Secondary
Poaceae	Wheat	*Triticum aestivum* L.	*Aegilops geniculata* Roth	Secondary
Poaceae	Wheat	*Triticum aestivum* L.	*Aegilops kotschyi* Boiss.	Secondary
Poaceae	Wheat	*Triticum aestivum* L.	*Aegilops longissima* Schweinf. et Muschl.	Secondary
Poaceae	Wheat	*Triticum aestivum* L.	*Aegilops markgrafii* (Greuter) K.Hammer	Secondary
Poaceae	Wheat	*Triticum aestivum* L.	*Aegilops neglecta* Req. ex Bertol.	Secondary
Poaceae	Wheat	*Triticum aestivum* L.	*Aegilops peregrina* (Hack.) Maire et Weiller	Secondary
Poaceae	Wheat	*Triticum aestivum* L.	*Aegilops peregrina* (Hack.) Maire et Weiller var. *brachyathera* (Boiss.) Maire et Weiller	Secondary
Poaceae	Wheat	*Triticum aestivum* L.	*Aegilops peregrina* (Hack.) Maire et Weiller var. *peregrina*	Secondary
Poaceae	Wheat	*Triticum aestivum* L.	*Aegilops sharonensis* Eig	Secondary
Poaceae	Wheat	*Triticum aestivum* L.	*Aegilops speltoides* Tausch	Secondary
Poaceae	Wheat	*Triticum aestivum* L.	*Aegilops tauschii* Coss.	Secondary
Poaceae	Wheat	*Triticum aestivum* L.	*Aegilops triaristata* Willd.	Secondary
Poaceae	Wheat	*Triticum aestivum* L.	*Aegilops triuncialis* L.	Secondary
Poaceae	Wheat	*Triticum aestivum* L.	*Aegilops triuncialis* L. var. *triuncialis*	Secondary
Poaceae	Wheat	*Triticum aestivum* L.	*Aegilops umbellulata* Zhuk.	Secondary
Poaceae	Wheat	*Triticum aestivum* L.	*Aegilops ventricosa* Tausch	Secondary
Poaceae	Wheat	*Triticum aestivum* L.	*Agropyron cristatum* (L.) Beauv.	Tertiary
Poaceae	Wheat	*Triticum aestivum* L.	*Agropyron cristatum* (L.) Beauv. subsp. *cristatum*	Tertiary
Poaceae	Wheat	*Triticum aestivum* L.	*Agropyron cristatum* (L.) Beauv. subsp. *pectinatum* (M. Bieb.) Tzvelev	Tertiary
Amaryllidaceae	Onion	*Allium cepa* L.	*Allium albidum* C.Presl.	Secondary
Amaryllidaceae	Leek	*Allium porrum* L.	*Allium altoatlanticum* Seregin	Tertiary
Amaryllidaceae	Garlic	*Allium cepa* L.	*Allium ampeloprasum* L.	Primary
Amaryllidaceae	Garlic	*Allium cepa* L.	*Allium ampeloprasum* L. var. *ampeloprasum*	Primary

Family	Crop	Crop Latin Name	CWR	Genepool
Amaryllidaceae	Onion	*Allium cepa* L.	*Allium atroviolaceum* Boiss.	Secondary
Amaryllidaceae	Onion	*Allium cepa* L.	*Allium bourgeaui* Rech.f.	Secondary
Amaryllidaceae	Onion	*Allium cepa* L.	*Allium commutatum* Guss.	Secondary
Amaryllidaceae	Onion	*Allium cepa* L.	*Allium longicuspis* Regel	Secondary
Amaryllidaceae	Garlic	*Allium sativum* L.	*Allium porrum* L.	Tertiary
Amaryllidaceae	Onion	*Allium cepa* L.	*Allium scabriscapum* Boiss.	Secondary
Amaryllidaceae	Onion	*Allium cepa* L.	*Allium schoenoprasum* L.	Primary
Amaryllidaceae	Onion	*Allium cepa* L.	*Allium sinaiticum* Boiss.	Tertiary
Amaryllidaceae	Onion	*Allium cepa* L.	*Allium sphaerocephalon* L.	Tertiary
Amaryllidaceae	Onion	*Allium cepa* L.	*Allium valdecallosum* Maire et Weiller	Tertiary
Poaceae	Wheat	*Triticum aestivum* L.	*Amblyopyrum muticum* (Boiss.) Eig.	Tertiary
Apiaceae	Celery	*Apium gravelens* L.	*Apium repens* Jacq.	Tertiary
Asparagaceae	Asparagus	*Asparagus officinalis* L.	*Asparagus acutifolius* L.	Secondary
Asparagaceae	Asparagus	*Asparagus officinalis* L.	*Asparagus albus* L.	Secondary
Asparagaceae	Asparagus	*Asparagus officinalis* L.	*Asparagus aphyllus* L.	Secondary
Asparagaceae	Asparagus	*Asparagus officinalis* L.	*Asparagus horridus* L.	Secondary
Asparagaceae	Asparagus	*Asparagus officinalis* L.	*Asparagus maritimus* (L.) Miller	Secondary
Asparagaceae	Asparagus	*Asparagus officinalis* L.	*Asparagus officinalis* L.	Primary
Asparagaceae	Asparagus	*Asparagus officinalis* L.	*Asparagus pastorianus* Webb et Berthel.	Secondary
Asparagaceae	Asparagus	*Asparagus officinalis* L.	*Asparagus tenuifolius* L.	Secondary
Asparagaceae	Asparagus	*Asparagus officinalis* L.	*Asparagus verticillatus* L.	Secondary
Poaceae	Oat	*Avena sativa* L.	*Avena agadiriana* B.R.Baum et G.Fedak	Secondary
Poaceae	Oat	*Avena sativa* L.	*Avena atlantica* B.R.Baum et G.Fedak	Tertiary
Poaceae	Oat	*Avena sativa* L.	*Avena barbata* Pott ex Link	Tertiary
Poaceae	Oat	*Avena sativa* L.	*Avena clauda* Durieu	Tertiary
Poaceae	Oat	*Avena sativa* L.	*Avena damascena* Rajh. et B.R.Baum	Tertiary
Poaceae	Oat	*Avena sativa* L.	*Avena eriantha Durieu*	Tertiary
Poaceae	Oat	*Avena sativa* L.	*Avena fatua* L.	Primary
Poaceae	Oat	*Avena sativa* L.	*Avena hybrida*	Tertiary
Poaceae	Oat	*Avena sativa* L.	*Avena longiglumis* Durieu	Tertiary
Poaceae	Oat	*Avena sativa* L.	*Avena macrostachya* Balansa et Durieu	Tertiary
Poaceae	Oat	*Avena sativa* L.	*Avena magna* H.C.Murphy et Terrell	Secondary
Poaceae	Oat	*Avena sativa* L.	*Avena matritensis* B.R.Baum	Tertiary

**Family**	**Crop**	**Crop Latin Name**	**CWR**	**Genepool**
Poaceae	Oat	*Avena sativa* L.	*Avena murphyi* Ladiz.	Secondary
Poaceae	Oat	*Avena sativa* L.	*Avena occidentalis* Durieu	Primary
Poaceae	Oat	*Avena sativa* L.	*Avena sterilis* L.	Primary
Poaceae	Oat	*Avena sativa* L.	*Avena trichophylla* K.Koch	Primary
Poaceae	Oat	*Avena sativa* L.	*Avena ventricosa* Balansa ex Coss.	Tertiary
Poaceae	Oat	*Avena sativa* L.	*Avena wiestii* Steud.	Tertiary
Chenopodiaceae	Sugar Beet	*Beta vulgaris* L.	*Beta corolliflora* Zossimovic ex Buttler	Tertiary
Chenopodiaceae	Sugar Beet	*Beta vulgaris* L.	*Beta intermedia* L.	Secondary
Chenopodiaceae	Sugar Beet	*Beta vulgaris* L.	*Beta lomatogona* Fisch. & C.A.May.	Tertiary
Chenopodiaceae	Sugar Beet	*Beta vulgaris* L.	*Beta macrocarpa* Guss.	Primary
Chenopodiaceae	Sugar Beet	*Beta vulgaris* L.	*Beta macrorhiza* Stev.	Tertiary
Chenopodiaceae	Sugar Beet	*Beta vulgaris* L.	*Beta trigyna* Walst. & Kit.	Tertiary
Chenopodiaceae	Sugar Beet	*Beta vulgaris* L.	*Beta vulgaris* L.	Primary
Chenopodiaceae	Sugar Beet	*Beta vulgaris* L.	*Beta vulgaris*subsp. *maritima* (L.) Arcang.	Primary
Amaranthaceae	Spinach	Spinacia oleracea L.	*Blitum virgatum* Link.	Tertiary
Brassicaceae	Turnip	*Brassica rapa* L.	*Brassica barrelieri* (L.) Janka	Secondary
Brassicaceae	Rapeseed	*Brassica napus* L.	*Brassica deserti* Danin et Hedge	Tertiary
Brassicaceae	Rapeseed	*Brassica napus* L.	*Brassica desnottesii* Emb. et Maire	Tertiary
Brassicaceae	Rapeseed	*Brassica napus* L.	*Brassica dimorpha* Coss. et Durieu	Tertiary
Brassicaceae	Cabbage	*Brassica oleracea* L.	*Brassica elongata* Ehrh.	Secondary
Brassicaceae	Cabbage	*Brassica oleracea* L.	*Brassica elongata* Ehrh. subsp. *imdrhasiana* Quezel	Secondary
Brassicaceae	Cabbage	*Brassica oleracea* L.	*Brassica elongata* Ehrh. subsp. *subscaposa* (Maire et Weiller) Maire	Secondary
Brassicaceae	Turnip	*Brassica rapa* L.	*Brassica fruticulosa* Cirillo	Tertiary
Brassicaceae	Rapeseed	*Brassica napus* L.	*Brassica fruticulosa* Cirillo subsp. *cossoniana* (Boiss. et Reut.) Maire	Tertiary
Brassicaceae	Rapeseed	*Brassica napus* L.	*Brassica fruticulosa* Cirillo subsp. *glaberrima* (Pomel) Batt.	Tertiary
Brassicaceae	Turnip	*Brassica rapa* L.	*Brassica fruticulosa* Cirillo subsp. *mauritanica* (Coss.) Maire	Tertiary
Brassicaceae	Rapeseed	*Brassica napus* L.	*Brassica fruticulosa* Cirillo subsp. *numidica* (Coss.) Maire	Tertiary
Brassicaceae	Turnip	*Brassica rapa* L.	*Brassica fruticulosa* Cirillo subsp. *pomeliana* Maire	Tertiary
Brassicaceae	Turnip	*Brassica rapa* L.	*Brassica fruticulosa* Cirillo subsp. *radicata* (Desf.) Batt.	Tertiary
Brassicaceae	Cabbage	*Brassica oleracea* L.	*Brassica gravinae* Ten.	Tertiary
Brassicaceae	Cabbage	*Brassica oleracea* L.	*Brassica insularis* Moris	Secondary
Brassicaceae	Cabbage	*Brassica oleracea* L.	*Brassica maurorum* Durieu	Secondary
Brassicaceae	Black Mustard	*Brassica juncea* (L.) Czern.	*Brassica nigra* (L.) W.D.J.Koch	Primary

**Family**	**Crop**	**Crop Latin Name**	**CWR**	**Genepool**
Brassicaceae	Black Mustard	*Brassica juncea* (L.) Czern.	*Brassica oxyrrhina* (Coss.) Willk.	Tertiary
Brassicaceae	Rapeseed	*Brassica napus* L.	*Brassica rapa* L.	Secondary
Brassicaceae	Turnip	*Brassica rapa* L.	*Brassica rapa*L. subsp. *oleifera* (DC.) Metzg	Primary
Brassicaceae	Rapeseed	*Brassica napus* L.	*Brassica repanda* (Willd.) DC.	Tertiary
Brassicaceae	Rapeseed	*Brassica napus* L.	*Brassica souliei* Batt. subsp. *amplexicaulis* (Desf.) Greuter et Burdet	Tertiary
Brassicaceae	Turnip	*Brassica rapa* L.	*Brassica spinescens* Pomel	Tertiary
Brassicaceae	Cabbage	*Brassica oleracea* L.	*Brassica tournefortii* Gouan	Secondary
Brassicaceae	Cabbage	*Brassica carinata* A.Braun	*Camelina sativa* (L.) Crantz	Tertiary
Brassicaceae	Turnip	*Brassica rapa* L.	*Capsella bursa-pastoris* (L.) Medik.	Tertiary
Brassicaceae	Cabbage	*Brassica oleracea* L.	*Capsella bursa-pastoris* (L.) Medik.	Tertiary
Brassicaceae	Rapeseed	*Brassica napus* L.	*Capsella bursa-pastoris* (L.) Medik.	Tertiary
Cyperaceae	Forage sedge	*Carex physodea* M.Bieb.	*Carex elata* All.	Tertiary
Cyperaceae	Forage sedge	*Carex physodea* M.Bieb.	*Carex illegitima* Ces.	Tertiary
Cyperaceae	Forage sedge	*Carex physodea* M.Bieb.	*Carex maritima* Gunnerus	Tertiary
Cyperaceae	Forage sedge	*Carex physodea* M.Bieb.	*Carex paniculata* L.	Tertiary
Cyperaceae	Forage sedge	*Carex physodea* M.Bieb.	*Carex pseudocyperus* L.	Tertiary
Cyperaceae	Forage sedge	*Carex physodea* M.Bieb.	*Carex riparia* Curtis	Tertiary
Asteraceae	Safflower	*Carthamus tinctorius* L.	*Carthamus alexandrinus* (Boiss. et Heldr.) Asch.	Tertiary
Asteraceae	Safflower	*Carthamus tinctorius* L.	*Carthamus creticus* L.	Secondary
Asteraceae	Safflower	*Carthamus tinctorius* L.	*Carthamus curdicus* Hanelt	Tertiary
Asteraceae	Safflower	*Carthamus tinctorius* L.	*Carthamus dentatus* Vahl	Tertiary
Asteraceae	Safflower	*Carthamus tinctorius* L.	*Carthamus divaricatus* Beg. et Vacc.	Secondary
Asteraceae	Safflower	*Carthamus tinctorius* L.	*Carthamus glaucus* M.Bieb.	Tertiary
Asteraceae	Safflower	*Carthamus tinctorius* L.	*Carthamus glaucus* M.Bieb. subsp. *alexandrinus* (Boiss. et Heldr.) Hanelt	Tertiary
Asteraceae	Safflower	*Carthamus tinctorius* L.	*Carthamus lanatus* L.	Tertiary
Asteraceae	Safflower	*Carthamus tinctorius* L.	*Carthamus lanatus* L. subsp. *montanus* (Pomel) Jahand. et Maire	Tertiary
Asteraceae	Safflower	*Carthamus tinctorius* L.	*Carthamus nitidus* Boiss.	Tertiary
Asteraceae	Safflower	*Carthamus tinctorius* L.	*Carthamus oxyacantha* M.Bieb.	Tertiary
Asteraceae	Safflower	*Carthamus tinctorius* L.	*Carthamus persicus* Desf. ex Willd.	Tertiary
Asteraceae	Safflower	*Carthamus tinctorius* L.	*Carthamus tenuis* (Boiss. & C.I.Blanche) Bornm.	Tertiary
Asteraceae	Safflower	*Carthamus tinctorius* L.	*Carthamus turkestanicus* Popov	Tertiary
Apiaceae	Caraway	*Carum carvi* L.	*Carum asinorum* Litard. et Maire	Tertiary
Apiaceae	Caraway	*Carum carvi* L.	*Carum carvi* L.	Primary

**Family**	**Crop**	**Crop Latin Name**	**CWR**	**Genepool**
Apiaceae	Caraway	*Carum carvi* L.	*Carum lacuum* Emb.	Tertiary
Fagaceae	Sweet Chestnut	*Castanea sativa* Mill.	*Castanea sativa* Mill.	Primary
Fabaceae	Carob	*Ceratonia siliqua* L.	*Ceratonia siliqua* L.	Primary
Chenopodiaceae	Quinoa	*Chenopodium quinoa* Willd.	*Chenopodium album* L.	Secondary
Chenopodiaceae	Quinoa	*Chenopodium quinoa* Willd.	*Chenopodium ficifolium* Sm.	Secondary
Chenopodiaceae	Quinoa	*Chenopodium quinoa* Willd.	*Chenopodium murale* L.	Tertiary
Chenopodiaceae	Quinoa	*Chenopodium quinoa* Willd.	*Chenopodium opulifolium* Schrad. ex W.D.J.Koch et Ziz	Tertiary
Chenopodiaceae	Quinoa	*Chenopodium quinoa* Willd.	*Chenopodium urbicum* L.	Tertiary
Chenopodiaceae	Quinoa	*Chenopodium quinoa* Willd.	*Chenopodium vulvaria* L.	Tertiary
Fabaceae	Chickpea	*Cicer arietinum* L.	*Cicer atlanticum* Coss. ex Maire	Tertiary
Fabaceae	Chickpea	*Cicer arientenum* L.	*Cicer bijugum* Rech.f.	Primary
Fabaceae	Chickpea	*Cicer arietinum* L.	*Cicer cuneatum* Hochst. ex A.Rich.	Tertiary
Fabaceae	Chickpea	*Cicer arientenum* L.	*Cicer echinospermum* P.H.Davis	Primary
Fabaceae	Chickpea	*Cicer arientenum* L.	*Cicer incisum* (Willd.) K.Malý	Secondary
Fabaceae	Chickpea	*Cicer arientenum* L.	*Cicer judaicum* Boiss.	Secondary
Fabaceae	Chickpea	*Cicer arientenum* L.	*Cicer pinnatifidum* Jaub. & Spach.	Secondary
Fabaceae	Chickpea	*Cicer arientenum* L.	*Cicer reticulatum* Ladiz.	Primary
Asteraceae	Endive	*Cichorium endivia* L.	*Cichorium calvum* Asch.	Primary
Asteraceae	Chicory	*Cichorium intybus* L.	*Cichorium glandulosum* Jacq.	Secondary
Asteraceae	Chicory	*Cichorium intybus* L.	*Cichorium intybus* L.	Primary
Asteraceae	Endive	*Cichorium endivia* L.	*Cichorium pumilum* Jacq.	Primary
Asteraceae	Chicory	*Cichorium intybus* L.	*Cichorium spinosum* L.	Primary
Cucurbitaceae	Watermelon	*Citrullus lanatus* (Thunb.) Matsum. et Nakai	*Citrullus colocynthis* (L.) Schrad.	Secondary
Cucurbitaceae	Watermelon	*Citrullus lanatus* (Thunb.) Matsum. et Nakai	*Citrullus lanatus* (Thunb.) Matsum. et Nakai	Primary
Betulaceae	Hazel	*Corylus avellana* L.	*Corylus avellana* L.	Primary
Brassicaceae	Crambe	*Crambe hispanica* L.	*Crambe filiformis* Jacq.	Secondary
Brassicaceae	Crambe	*Crambe hispanica* L.	*Crambe glabrata* DC.	Tertiary
Brassicaceae	Cabbage	*Brassica oleracea* L.	*Crambe hispanica* L. subsp. *hispanica*	Primary
Brassicaceae	Crambe	*Crambe hispanica* L.	*Crambe kralikii* Coss.	Tertiary
Cucurbitaceae	Melon	*Cucumis melo* L.	*Cucumis dipsaceus* Ehrenb. ex Spach	Tertiary
Cucurbitaceae	Melon	*Cucumis melo* L.	*Cucumis prophetarum* L.	Secondary

**Family**	**Crop**	**Crop Latin Name**	**CWR**	**Genepool**
Asteraceae	Artichoke	*Cynara algarbiensis* Coss. ex Mariz	*Cynara baetica* (Spreng.) Pau	Secondary
Asteraceae	Artichoke	*Cynara algarbiensis* Coss. ex Mariz	*Cynara baetica* Wiklund subsp. *maroccana* Wiklund	Secondary
Asteraceae	Artichoke	*Cynara algarbiensis* Coss. ex Mariz	*Cynara cardunculus* Wiklund subsp. *flavescens* Wiklund	Primary
Asteraceae	Artichoke	*Cynara algarbiensis* Coss. ex Mariz	*Cynara cornigera* Lindley	Tertiary
Asteraceae	Artichoke	*Cynara algarbiensis* Coss. ex Mariz	*Cynara cyrenaica* Maire et Weiller	Tertiary
Asteraceae	Artichoke	*Cynara algarbiensis* Coss. ex Mariz	*Cynara humilis* L.	Secondary
Asteraceae	Artichoke	*Cynara algarbiensis* Coss. ex Mariz	*Cynara tournefortii* Boiss. et Reut.	Secondary
Poaceae	Wheat	*Triticum aestivum* L.	*Dasypyrum villosum* (L.) Borbás	Primary
Apiaceae	Carrot	*Daucus carota* L.	*Daucus aureus* Desf.	Secondary
Apiaceae	Carrot	*Daucus carota* L.	*Daucus capillifolius* Gilli	Primary
Apiaceae	Carrot	*Daucus carota* L.	*Daucus carota* L.	Primary
Apiaceae	Carrot	*Daucus carota* L.	*Daucus carota* L. subsp. *capillifolius* (Gilli) Arbizu	Primary
Apiaceae	Carrot	*Daucus carota* L.	*Daucus carota* L. subsp. *azoricus* Franco	Primary
Apiaceae	Carrot	*Daucus carota* L.	*Daucus carota* L. subsp. *carota* carota	Primary
Apiaceae	Carrot	*Daucus carota* L.	*Daucus carota* L. subsp. *drepanensis* (Arcang.) Heywood	Primary
Apiaceae	Carrot	*Daucus carota* L.	*Daucus carota* L. subsp. *fontanesii* Thell.	Primary
Apiaceae	Carrot	*Daucus carota* L.	*Daucus carota* L. subsp. *gummifer* (Syme) Hook. f.	Primary
Apiaceae	Carrot	*Daucus carota* L.	*Daucus carota* L. subsp. *hispanicus* (Gouan) Thell.	Primary
Apiaceae	Carrot	*Daucus carota* L.	*Daucus carota* L. subsp. *maritimus* (Lam.) Batt	Primary
Apiaceae	Carrot	*Daucus carota* L.	*Daucus carota* L. subsp. *maximus* (Desf.) Ball	Primary
Apiaceae	Carrot	*Daucus carota* L.	*Daucus carota* L. subsp. *parviflorus* (Desf.) Thell.	Primary
Apiaceae	Carrot	*Daucus carota* L.	*Daucus crinitus* Desf.	Tertiary
Apiaceae	Carrot	*Daucus carota* L.	*Daucus gracilis* Steinh.	Secondary
Apiaceae	Carrot	*Daucus carota* L.	*Daucus jordanicus* Post	Secondary
Apiaceae	Carrot	*Daucus carota* L.	*Daucus muricatus* (L.) L.	Secondary
Apiaceae	Carrot	*Daucus carota* L.	*Daucus sahariensis* Murb.	Secondary
Apiaceae	Carrot	*Daucus carota* L.	*Daucus syrticus* Murb.	Secondary
Apiaceae	Carrot	*Daucus carota* L.	*Daucus tenuisectus* Coss. ex Batt.	Tertiary
Brassicaceae	Rapeseed	*Brassica napus* L.	*Descurainia sophia* (L.) Webb ex Prantl	Tertiary
Poaceae	Crabgrass	*Digitaria exilis* (Kippist) Stapf	*Digitaria debilis* (Desf.) Willd.	Tertiary
Poaceae	Crabgrass	*Digitaria exilis* (Kippist) Stapf	*Digitaria nodosa* Parl.	Tertiary
Poaceae	Crabgrass	*Digitaria exilis* (Kippist) Stapf	*Digitaria sanguinalis* (L.) Scop.	Tertiary
Poaceae	Crabgrass	*Digitaria exilis* (Kippist) Stapf	*Digitaria velutina* (Forssk.) P. Beauv.	Tertiary

Family	Crop	Crop Latin Name	CWR	Genepool
Brassicaceae	Rapeseed	*Brassica napus* L.	*Diplotaxis acris* (Forsk.) Boiss.	Tertiary
Brassicaceae	Cabbage	*Brassica carinata* A.Braun	*Diplotaxis assurgens* (Del.) Gren.	Tertiary
Brassicaceae	Mustard	*Brassica juncea* (L.) Czern.	*Diplotaxis berthautii* Braun-Blanq. et Maire	Tertiary
Brassicaceae	Rapeseed	*Brassica napus* L.	*Diplotaxis catholica* (L.) DC.	Tertiary
Brassicaceae	Turnip	*Brassica rapa* L.	*Diplotaxis erucoides* (L.) DC.	Tertiary
Brassicaceae	Mustard	*Brassica juncea* (L.) Czern.	*Diplotaxis harra* (Forssk.) Boiss.	Tertiary
Brassicaceae	Black Mustard	*Brassica nigra* (L.) W.D.J.Koch	*Diplotaxis maurorum* (Durieu) M.B.Crespo et M.Fabregat	Secondary
Brassicaceae	Turnip	*Brassica rapa* L.	*Diplotaxis muralis* (L.) DC.	Tertiary
Brassicaceae	Rapeseed	*Brassica napus* L.	*Diplotaxis siifolia* Kunze	Tertiary
Brassicaceae	Arugula	*Eruca vesicaria* (L.) Cav.	*Diplotaxis simplex* (Viv.) Spreng.	Secondary
Brassicaceae	Arugula	*Eruca vesicaria* (L.) Cav.	*Diplotaxis tenuifolia* (L.) DC.	Secondary
Brassicaceae	Rapeseed	*Brassica napus* L.	*Diplotaxis viminea* (L.) DC.	Tertiary
Brassicaceae	Turnip	*Brassica rapa* L.	*Diplotaxis virgata* (Cav.) DC.	Tertiary
Rosaceae	Strawberries	*Fragaria x ananassa* Duchesne ex Rozier	*Drymocallis rupestris* (L.) Sojak	Tertiary
Poaceae	Wheat	*Triticum aestivum* L.	*Elymus caninus L.*	Tertiary
Poaceae	Wheat	*Triticum aestivum* L.	*Elymus panormitanus* (Parl.) Tzvelev	Tertiary
Poaceae	Wheat	*Triticum aestivum* L.	*Elymus repens* (L.) Gould	Tertiary
Poaceae	Wheat	*Triticum aestivum* L.	*Elymus repens* (L.) Gould subsp. *repens*	Tertiary
Brassicaceae	Cabbage	*Brassica carinata* A. Braun	*Enarthrocarpus lyratus* (Forssk.) DC.	Tertiary
Brassicaceae	Arugula	*Eruca vesicaria* (L.) Cav.	*Eruca vesicaria* (L.) Cav. subsp. *pinnatifida* (Desf.) Thell.	Primary
Brassicaceae	Arugula	*Eruca vesicaria* (L.) Cav.	*Eruca vesicaria* (L.) Cav. subsp. *sativa* (Mill.) Thell.	Primary
Brassicaceae	Arugula	*Eruca vesicaria* (L.) Cav.	*Eruca vesicaria* (L.) Cav. subsp. *vesicaria* (L.) Cav.	Primary
Moraceae	Fig	*Ficus carica* L.	*Ficus carica* L. subsp. *rupestris* (Hausskn. ex Boiss.) Browicz	Primary
Moraceae	Fig	*Ficus carica* L.	*Ficus palmata* Forssk.	Secondary
Rosaceae	Strawberries	*Fragaria x ananassa* Duchesne ex Rozier	*Fragaria vesca* L.	Tertiary
Rosaceae	Strawberries	*Fragaria x ananassa* Duchesne ex Rozier	*Fragaria viridis* Breslau.	Primary
Brassicaceae	Black Mustard	*Brassica nigra* (L.) W.D.J.Koch	*Hirschfeldia incana* (L.) Lagr. Foss.	Tertiary
Poaceae	Barley	*Hordeum vulgare* L.	*Hordeum brevisubulatum* (Trin.) Link	Secondary
Poaceae	Barley	*Hordeum vulgare* L.	*Hordeum bulbosum* L.	Secondary
Poaceae	Barley	*Hordeum vulgare* L.	*Hordeum marinum* (Parl.) Thell. subsp. *gussoneanum* (Parl.) Thell.	Tertiary

**Family**	**Crop**	**Crop Latin Name**	**CWR**	**Genepool**
Poaceae	Barley	*Hordeum vulgare* L.	*Hordeum marinum* Huds.	Tertiary
Poaceae	Barley	*Hordeum vulgare* L.	*Hordeum marinum* Huds. subsp. *marinum*	Tertiary
Poaceae	Barley	*Hordeum vulgare* L.	*Hordeum murinum* L. subsp. *leporinum* (Link) Arcang.	Tertiary
Poaceae	Barley	*Hordeum vulgare* L.	*Hordeum murinum* L. subsp. *glaucum* (Steud.) Tzvelev	Tertiary
Poaceae	Barley	*Hordeum vulgare* L.	*Hordeum murinum* L.	Tertiary
Poaceae	Barley	*Hordeum vulgare* L.	*Hordeum secalinum* Schreber	Tertiary
Poaceae	Barley	*Hordeum vulgare* L.	*Hordeum vulgare* L.	Primary
Poaceae	Barley	*Hordeum vulgare* L.	*Hordeum vulgare* L. subsp. *spontaneum* (C.Koch.) Thell.	Primary
Cannabaceae	Hop	*Humulus lupulus* L.	*Humulus lupulus* L. subsp. *lupulus*	Primary
Poaceae	Sugar Cane	*Saccharum officinarum* L.	*Imperata cylindrica* (L.) P.Beauv.	Tertiary
Brassicaceae	Woad	*Isatis tinctoria* L.	*Isatis lusitanica* L.	Secondary
Brassicaceae	Woad	*Isatis tinctoria* L.	*Isatis microcarpa* Boiss.	Tertiary
Brassicaceae	Woad	*Isatis tinctoria* L.	*Isatis tinctoria* L.	Tertiary
Juglandaceae	English Walnut	*Juglans major* L.	*Juglans regia* L.	Primary
Asteraceae	Lettuce	*Lactuca sativa* L.	*Lactuca aculeata* Boiss.	Tertiary
Asteraceae	Lettuce	*Lactuca sativa* L.	*Lactuca orientalis* (Boiss.) Boiss.	Tertiary
Asteraceae	Lettuce	*Lactuca sativa* L.	*Lactuca quercina* L.	Tertiary
Asteraceae	Lettuce	*Lactuca sativa* L.	*Lactuca saligna* L.	Secondary
Asteraceae	Lettuce	*Lactuca sativa* L.	*Lactuca serriola* L.	Primary
Asteraceae	Lettuce	*Lactuca sativa* L.	*Lactuca tatarica* (L.) C.A.Mey.	Tertiary
Asteraceae	Lettuce	*Lactuca sativa* L.	*Lactuca viminea* (L.) J.Presl et C.Presl	Tertiary
Asteraceae	Lettuce	*Lactuca sativa* L.	*Lactuca viminea* (L.) J.Presl et C.Presl subsp. *chondrilliflora* (Boreau) St.-Lag.	Tertiary
Asteraceae	Lettuce	*Lactuca sativa* L.	*Lactuca viminea* (L.) J.Presl et C.Presl subsp. *ramosissima* (All.) Arcang.	Tertiary
Asteraceae	Lettuce	*Lactuca sativa* L.	*Lactuca viminea* (L.) J.Presl et C.Presl subsp. *viminea*	Tertiary
Asteraceae	Lettuce	*Lactuca sativa* L.	*Lactuca virosa* L.	Tertiary
Asteraceae	Lettuce	*Lactuca sativa* L.	*Lactuca virosa* L. subsp. *cornigera* (Pau et Font Quer) Emb. et Maire	Tertiary
Asteraceae	Lettuce	*Lactuca sativa* L.	*Lactuca virosa* L. subsp. *virosa*	Tertiary
Fabaceae	Grasspea	*Lathyrus sativus* L.	*Lathyrus amphicarpos* L.	Secondary
Fabaceae	Grasspea	*Lathyrus sativus* L.	*Lathyrus annuus* L.	Tertiary
Fabaceae	Grasspea	*Lathyrus sativus* L.	*Lathyrus cicera* L.	Secondary
Fabaceae	Grasspea	*Lathyrus ochrus* (L.) DC.	*Lathyrus clymenum* L.	Tertiary
Fabaceae	Grasspea	*Lathyrus sativus* L.	*Lathyrus gorgoni* Parl.	Tertiary
Fabaceae	Grasspea	*Lathyrus sativus* L.	*Lathyrus hierosolymitanus* Boiss.	Tertiary

**Family**	**Crop**	**Crop Latin Name**	**CWR**	**Genepool**
Fabaceae	Grasspea	*Lathyrus sativus* L.	*Lathyrus hirsutus* L.	Tertiary
Fabaceae	Sweet Pea	*Lathyrus odoratus* L.	*Lathyrus latifolius* L.	Secondary
Fabaceae	Grasspea	*Lathyrus sativus* L.	*Lathyrus marmoratus* Boiss. et Blanche	Tertiary
Fabaceae	Grasspea	*Lathyrus ochrus* (L.) DC.	*Lathyrus ochrus* (L.) DC.	Primary
Fabaceae	Grasspea	*Lathyrus sativus* L.	*Lathyrus pseudocicera* Pamp.	Tertiary
Fabaceae	Sweet Pea	*Lathyrus odoratus* L.	*Lathyrus sylvestris* L.	Secondary
Fabaceae	Grasspea	*Lathyrus sativus* L.	*Lathyrus tingitanus* L.	Tertiary
Fabaceae	Lentil	*Lens culinaris* Medik.	*Lens culinaris* Medik. subsp. *odemensis* (Ladiz.) M.E.Ferguson et al.	Primary
Fabaceae	Lentil	*Lens culinaris* Medik.	*Lens culinaris* Medik. subsp. *orientalis*	Primary
Fabaceae	Lentil	*Lens culinaris* Medik.	*Lens ervoides* (Brign.) Grande	Secondary
Fabaceae	Lentil	*Lens culinaris* Medik.	*Lens lamottei* Czefr.	Secondary
Fabaceae	Lentil	*Lens culinaris* Medik.	*Lens nigricans* (M.Bieb.) Godr.	Secondary
Poaceae	Rye	*Secale cereale* L.	*Leymus racemosus* (Lam.) Tzvelev	Secondary
Linaceae	Flax	*Linum usitatissimum* L.	*Linum austriacum* (Pomel) Greuter et Burdet	Tertiary
Linaceae	Flax	*Linum usitatissimum* L.	*Linum austriacum* L.	Tertiary
Linaceae	Flax	*Linum usitatissimum* L.	*Linum bienne* Mill.	Primary
Linaceae	Flax	*Linum usitatissimum* L.	*Linum corymbiferum* Desf.	Secondary
Linaceae	Flax	*Linum usitatissimum* L.	*Linum decumbens* Desf.	Secondary
Linaceae	Flax	*Linum usitatissimum* L.	*Linum grandiflorum* Desf.	Tertiary
Linaceae	Flax	*Linum usitatissimum* L.	*Linum hirsutum* L.	Tertiary
Linaceae	Flax	*Linum usitatissimum* L.	*Linum hypericifolium* Salisb.	Tertiary
Linaceae	Flax	*Linum usitatissimum* L.	*Linum narbonense* L.	Tertiary
Linaceae	Flax	*Linum usitatissimum* L.	*Linum nervosum* Waldst. & Kit.	Tertiary
Linaceae	Flax	*Linum usitatissimum* L.	*Linum pubescens* Banks & Sol.	Tertiary
Fabaceae	Bird's foot trefoil	*Lotus corniculatus* L.	*Lotus benoistii* (Maire) Lassen	Tertiary
Fabaceae	Lupins	*Lupinus angustifolius* L.	*Lupinus albus* L.	Secondary
Fabaceae	Lupins	*Lupinus angustifolius* L.	*Lupinus angustifolius* L.	Primary
Fabaceae	Lupins	*Lupinus luteus* L.	*Lupinus atlanticus* Gladst.	Primary
Fabaceae	Lupins	*Lupinus cosentinii* Guss.	*Lupinus digitatus* Forssk.	Primary
Fabaceae	Lupins	*Lupinus cosentinii* Guss.	*Lupinus hispanicus* Boiss. et Reut.	Secondary
Fabaceae	Lupins	*Lupinus cosentinii* Guss.	*Lupinus luteus* L.	Primary
Fabaceae	Lupins	*Lupinus albus* L.	*Lupinus micranthus* Guss.	Secondary
Fabaceae	Lupins	*Lupinus cosentinii* Guss.	*Lupinus palaestinus* Boiss.	Secondary

Family	Crop	Crop Latin Name	CWR	Genepool
Apiaceae	Parsnip	*Pastinaca sativa* L. subsp. *sativa*	*Malabaila suaveolens* (Delile) Coss.	Tertiary
Rosaceae	Apple	*Malus domestica* Borkh.	*Malus florentina* (Zucc.) C.K.Schneid.	Secondary
Rosaceae	Apple	*Malus domestica* Borkh.	*Malus sylvestris* (L.) Mill.	Primary
Fabaceae	Alfalfa	*Medicago truncatula* Gaertn.	*Medicago doliata* Carmign.	Primary
Fabaceae	Alfalfa	*Medicago truncatula* Gaertn.	*Medicago italica* (Mill.) Fiori	Primary
Fabaceae	Alfalfa	*Medicago truncatula* Gaertn.	*Medicago lesinsii* F.Small	Tertiary
Fabaceae	Alfalfa	*Medicago truncatula* Gaertn.	*Medicago littoralis* Loisel.	Primary
Fabaceae	Alfalfa	*Medicago sativa* L.	*Medicago marina* L.	Tertiary
Fabaceae	Alfalfa	*Medicago truncatula* Gaertn.	*Medicago murex* Willd.	Tertiary
Fabaceae	Alfalfa	*Medicago truncatula* Gaertn.	*Medicago rigidula* (L.) All.	Secondary
Fabaceae	Alfalfa	*Medicago sativa* L.	*Medicago rugosa* Desr.	Potential
Fabaceae	Alfalfa	*Medicago sativa* L.	*Medicago sativa* L. subsp. *caerulea* (Less. ex Ledeb.) Schmalh.	Secondary
Fabaceae	Alfalfa	*Medicago sativa* L.	*Medicago sativa* L. subsp. *glomerata* (Balb.) Rouy	Primary
Fabaceae	Alfalfa	*Medicago sativa* L.	*Medicago sativa* L. subsp. *tunetana* Murb.	Primary
Fabaceae	Alfalfa	*Medicago sativa* L.	*Medicago sativa* L. var. *falcata* (L.) Arcang.	Primary
Fabaceae	Alfalfa	*Medicago truncatula* Gaertn.	*Medicago scutellata* (L.) Mill.	Potential
Fabaceae	Alfalfa	*Medicago truncatula* Gaertn.	*Medicago soleirolii* Duby	Primary
Fabaceae	Alfalfa	*Medicago truncatula* Gaertn.	*Medicago truncatula* Gaertn.	Primary
Fabaceae	Alfalfa	*Medicago truncatula* Gaertn.	*Medicago turbinata* (L.) All.	Primary
Lamiaceae	Mint, Spear	*Mentha spicata* L.	*Mentha aquatica* L.	Secondary
Lamiaceae	Mint, Pepper	*Mentha piperita* L.	*Mentha aquatica* L. var. *aquatica*	Secondary
Lamiaceae	Mint, Pepper	*Mentha piperita* L.	*Mentha arvensis* L.	Tertiary
Lamiaceae	Mint, Pepper	*Mentha piperita* L.	*Mentha longifolia* (L.) L.	Secondary
Lamiaceae	Mint, Pepper	*Mentha piperita* L.	*Mentha spicata* L.	Tertiary
Lamiaceae	Mint, Pepper	*Mentha piperita* L.	*Mentha suaveolens* Ehrh.	Tertiary
Brassicaceae	Mustard	*Brassica juncea* (L.) Czern.	*Moricandia arvensis* (L.) DC.	Tertiary
Brassicaceae	Rapeseed	*Brassica napus* L.	*Moricandia nitens* (Viv.) E.A.Durand et Barratte	Tertiary
Oleaceae	Olive	*Olea europaea* L.	*Olea europaea* L. subsp. *cuspidata* (Wall. et G.Don) Cif.	Primary
Oleaceae	Olive	*Olea europaea* L.	*Olea europaea* L. subsp. *europaea*	Primary
Oleaceae	Olive	*Olea europaea* L.	*Olea europaea* L. subsp. *europaea* var. *sativa* L.	Primary
Oleaceae	Olive	*Olea europaea* L.	*Olea europaea* L. subsp. *laperrinei* (Batt. et Trab.) Cif.	Primary
Oleaceae	Olive	*Olea europaea* L.	*Olea europaea* L. subsp. *maroccana* (Greuter et Burdet) P.Vargas et al.	Secondary
Apiaceae	Parsnip	*Pastinaca sativa* L.	*Pastinaca armena* Fisch. & C.A.Mey.	Tertiary

Family	Crop	Crop Latin Name	CWR	Genepool
Apiaceae	Parsnip	*Pastinaca sativa* L.	*Pastinaca gelendostensis* (Yıld. & B.Selvi) Hand	Tertiary
Apiaceae	Parsnip	*Pastinaca sativa* L.	*Pastinaca glandulosa* Boiss. & Hausskn.	Tertiary
Apiaceae	Parsnip	*Pastinaca sativa* L.	*Pastinaca pimpinellifolia* M.Bieb.	Tertiary
Apiaceae	Parsnip	*Pastinaca sativa* L.	*Pastinaca sativa* L.	Primary
Apiaceae	Parsnip	*Pastinaca sativa* L.	*Pastinaca yildizii* Dirmenci	Tertiary
Apiaceae	Parsnip	*Pastinaca sativa* L.	*Pastinaca zozimoides* Fenzl	Tertiary
Chenopodiaceae	Sugar Beet	*Beta vulgaris* L.	*Patellifolia patellaris* (Moq.) A.J.Scott et al.	Tertiary
Arecaceae	Date	*Phoenix dactylifera* L.	*Phoenix humilis* Royle.	Secondary
Arecaceae	Cretan date palm	*Phoenix theophrasti* Greuter	*Phoenix theophrasti* Greuter	Primary
Anacardiaceae	Pistachio	*Pistacia vera* L.	*Pistacia atlantica* Desf.	Tertiary
Anacardiaceae	Pistachio	*Pistacia vera* L.	*Pistacia atlantica* Desf. subsp. *atlantica*	Secondary
Anacardiaceae	Pistachio	*Pistacia vera* L.	*Pistacia eurycarpa* Yalt.	Tertiary
Anacardiaceae	Pistachio	*Pistacia vera* L.	*Pistacia khinjuk* Stocks	Tertiary
Anacardiaceae	Pistachio	*Pistacia vera* L.	*Pistacia lentiscus* L.	Secondary
Anacardiaceae	Pistachio	*Pistacia vera* L.	*Pistacia saportae* Burnat	graftstock
Anacardiaceae	Pistachio	*Pistacia vera* L.	*Pistacia terebinthus* L.	Tertiary
Anacardiaceae	Pistachio	*Pistacia vera* L.	*Pistacia terebinthus* L. subsp. *terebinthus*	Secondary
Fabaceae	Pea	*Pisum sativum* L.	*Pisum fulvum* Sibth. et Sm.	Secondary
Fabaceae	Pea	*Pisum sativum* L.	*Pisum sativum* L. subsp. *elatius* (M.Bieb.) Asch. et Graebn.	Primary
Fabaceae	Pea	*Pisum sativum* L.	*Pisum sativum* L. subsp. *elatius* (M.Bieb.) Asch. et Graebn. var. *elatius* (M.Bieb.) Alef.	Primary
Fabaceae	Pea	*Pisum sativum* L.	*Pisum sativum* L. subsp. *elatius* (M.Bieb.) Asch. et Graebn. var. *pumilio* Meikle	Secondary
Rosaceae	Cherry	*Prunus avium* L.	*Prunus arabica L.*	Tertiary
Rosaceae	Cherry	*Prunus avium* L.	*Prunus argentea* L.	Tertiary
Rosaceae	Cherry	*Prunus avium* L.	*Prunus avium* L.	Tertiary
Rosaceae	Cherry	*Prunus avium* L.	*Prunus carduchorum* (Bornm.) Meikle	Tertiary
Rosaceae	Cherry	*Prunus avium* L.	*Prunus cerasifera* Ehrh.	Tertiary
Rosaceae	Cherry	*Prunus avium* L.	*Prunus cocomilia* Ten.	Tertiary
Rosaceae	Cherry	*Prunus avium* L.	*Prunus discolor* (Spach) C.K.Schneid.	Tertiary
Rosaceae	Almond	*Prunus dulcis* (Mill.) D.A.Webb	*Prunus dulcis* (Mill.) D.A.Webb	Primary
Rosaceae	Cherry	*Prunus avium* L.	*Prunus fenzliana* Fritsch	Tertiary
Rosaceae	Cherry	*Prunus avium* L.	*Prunus kotschyi* (Boiss. & Hohen. ex Spach) Meikle	Tertiary

Family	Crop	Crop Latin Name	CWR	Genepool
Rosaceae	Cherry	*Prunus avium* L.	*Prunus lusitanica* L.	Tertiary
Rosaceae	Cherry	*Prunus avium* L.	*Prunus lycioides* (Spach) C.K.Schneid.	Tertiary
Rosaceae	Cherry	*Prunus avium* L.	*Prunus mahaleb* L.	Primary
Rosaceae	Cherrie	*Prunus cerasus* L.	*Prunus mahaleb* L.	Secondary
Rosaceae	Cherry	*Prunus avium* L.	*Prunus microcarpa* C.A.Mey.	Tertiary
Rosaceae	Cherry	*Prunus avium* L.	*Prunus padus* L.	Tertiary
Rosaceae	Plum	*Prunus domestica* L.	*Prunus prostrata* Labill.	Secondary
Rosaceae	Cherry	*Prunus avium* L.	*Prunus spinosa* L.	Secondary
Rosaceae	Cherry	*Prunus avium* L.	*Prunus trichamygdalus* Hand.-Mazz.	Tertiary
Rosaceae	Plum	*Prunus domestica* L.	*Prunus ursina* Kotschy	Tertiary
Rosaceae	Almond	*Prunus dulcis* (Mill.) D.A.Webb	*Prunus webbii* (Spach) Fritsch	Tertiary
Apiaceae	Carrot	*Daucus carota* L.	*Pseudorlaya pumila* (L.) Grande	Tertiary
Rosaceae	Pear	*Pyrus communis* L.	*Pyrus communis* L.	Primary
Rosaceae	Pear	*Pyrus communis* L.	*Pyrus cossonii* Rehder	Primary
Rosaceae	Pear	*Pyrus communis* L.	*Pyrus elaeagrifolia* Pall.	Tertiary
Rosaceae	Pear	*Pyrus communis* L.	*Pyrus gharbiana* Trab.	Secondary
Rosaceae	Pear	*Pyrus communis* L.	*Pyrus mamorensis* Trab.	Secondary
Brassicaceae	Radish	*Raphanus sativus* L.	*Raphanus raphanistrum* L.	Primary
Brassicaceae	Radish	*Raphanus sativus* L.	*Raphanus raphanistrum* L. subsp. *landra* (Moretti ex DC.) Bonnier et Layens	Primary
Brassicaceae	Radish	*Raphanus sativus* L.	*Raphanus raphanistrum* L. subsp. *raphanistrum*	Primary
Brassicaceae	Rapeseed	*Brassica napus* L.	*Rapistrum rugosum* (L.) All.	Tertiary
Rhamnaceae	Sea Buckthorn	*Hippophae rhamnoides* L.	*Rhamnus serpyllacea* Greuter et Burdet	Secondary
Grossulariaceae	Currant	*Ribes nigrum* L.	*Ribes petraeum* Wulfen	Secondary
Grossulariaceae	Currant	*Ribes nigrum* L.	*Ribes uva-crispa* L.	Tertiary
Brassicaceae	Kinakrasse	*Rorippa indica* (L.) Hiern	*Rorippa amphibia* (L.) Besser	Tertiary
Brassicaceae	Kinakrasse	*Rorippa indica* (L.) Hiern	*Rorippa atlantica* Maire	Tertiary
Brassicaceae	Kinakrasse	*Rorippa indica* (L.) Hiern	*Rorippa hayanica* Maire	Tertiary
Brassicaceae	Rapeseed	*Brassica napus* L.	*Rorippa indica* (L.) Hiern	Tertiary
Brassicaceae	Kinakrasse	*Rorippa indica* (L.) Hiern	*Rorippa palustris* (L.) Besser	Tertiary
Rosaceae	Raspberries	*Rubus occidentalis* L.	*Rubus idaeus* L.	Secondary
Rosaceae	Raspberries	*Rubus idaeus* L.	*Rubus idaeus* L. subsp. *idaeus*	Primary
Polygonaceae	Docks	*Rumex patientia* L.	*Rumex algeriensis* Barratte et Murb. ex Murb.	Secondary
Polygonaceae	Docks	*Rumex patientia* L.	*Rumex tunetanus* Barratte et Murb. ex Murb.	Secondary

Family	Crop	Crop Latin Name	CWR	Genepool
Poaceae	Sugar Cane	*Saccharum officinarum* L.	*Saccharum ravennae* (L.) L.	Tertiary
Poaceae	Sugar Cane	*Saccharum officinarum* L.	*Saccharum spontaneum* L.	Secondary
Poaceae	Sugar Cane	*Saccharum officinarum* L.	*Saccharum spontaneum* L. subsp. *aegyptiacum* (Willd.) Hack.	Secondary
Poaceae	Sugar Cane	*Saccharum officinarum* L.	*Saccharum strictum* (Host) Spreng.	Tertiary
Poaceae	Rye	*Secale cereale* L.	*Secale cereale* L.	Primary
Poaceae	Rye	*Secale cereale* L.	*Secale strictum* (Presl.) Presl.	Secondary
Poaceae	Rye	*Secale cereale* L.	*Secale sylvestre* Host	Tertiary
Poaceae	Rye	*Secale cereale* L.	*Secale vavilovii* Grossh.	Tertiary
Brassicaceae	White Mustard	*Sinapis alba* L.	*Sinapis alba* L.	Primary
Brassicaceae	White Mustard	*Sinapis alba* L.	*Sinapis alba* L. subsp. *alba*	Primary
Brassicaceae	White Mustard	*Sinapis alba* L.	*Sinapis alba* L. subsp. *dissecta* (Lag.) Simonk.	Primary
Brassicaceae	White Mustard	*Sinapis alba* L.	*Sinapis alba* L. subsp. *mairei* (H.Lindb.) Maire	Primary
Brassicaceae	Black Mustard	*Brassica nigra* (L.) W.D.J.Koch	*Sinapis arvensis* L.	Secondary
Brassicaceae	White Mustard	*Sinapis alba* L.	*Sinapis flexuosa* Poir.	Primary
Brassicaceae	Radish	*Raphanus sativus* L.	*Sinapis pubescens* L.	Tertiary
Amaranthaceae	Spinach	Spinacia oleracea L.	*Spinacia tetrandra* Steven ex M.Bieb.	Primary
Poaceae	Wheat	*Triticum aestivum* L.	*Thinopyrum bessarabicum* (Săvul. & Rayss) Á.Löve	Tertiary
Poaceae	Wheat	*Triticum aestivum* L.	*Thinopyrum caespitosum* (K.Koch) Zhi W.Liu & R.R.-C.Wang	Tertiary
Poaceae	Wheat	*Triticum aestivum* L.	*Thinopyrum elongatum* (Host) D.R.Dewey	Tertiary
Poaceae	Wheat	*Triticum aestivum* L.	*Thinopyrum intermedium* (Host) Barkworth & D.R.Dewey	Tertiary
Poaceae	Wheat	*Triticum aestivum* L.	*Thinopyrum junceum* (L.) A.Love	Tertiary
Poaceae	Wheat	*Triticum aestivum* L.	*Thinopyrum podperae* (Nábelek) D.R.Dewey	Tertiary
Poaceae	Wheat	*Triticum aestivum* L.	*Thinopyrum ponticum* (Podp.) Barkworth & D.R.Dewey	Tertiary
Poaceae	Wheat	*Triticum aestivum* L.	*Thinopyrum pycnanthum* (Godr.) Barkworth	Tertiary
Poaceae	Wheat	*Triticum aestivum* L.	*Thinopyrum sartorii* (Boiss. & Heldr.) Á.Löve	Tertiary
Poaceae	Wheat	*Triticum aestivum* L.	*Thinopyrum scirpeum* (C. Presl) D.R.Dewey	Tertiary
Brassicaceae	Mustard	*Brassica juncea* (L.) Czern.	*Trachystoma ballii* O.E.Schulz	Tertiary
Fabaceae	Clover	*Trifolium pratense* L.	*Trifolium angustifolium* L.	Tertiary
Fabaceae	Clover	*Trifolium pratense* L.	*Trifolium angustifolium* L. var. *angustifolium*	Tertiary
Fabaceae	Clover	*Trifolium pratense* L.	*Trifolium angustifolium* L. var. *intermedium* Gibelli et Belli	Tertiary
Fabaceae	Clover	*Trifolium pratense* L.	*Trifolium arvense* L.	Tertiary
Fabaceae	Clover	*Trifolium pratense* L.	*Trifolium bocconei* Savi	Tertiary
Fabaceae	Clover	*Trifolium pratense* L.	*Trifolium bocconei* Savi var. *bocconei*	Tertiary

**Family**	**Crop**	**Crop Latin Name**	**CWR**	**Genepool**
Fabaceae	Clover	*Trifolium pratense* L.	*Trifolium cernuum* Brot.	Tertiary
Fabaceae	Clover	*Trifolium pratense* L.	*Trifolium cherleri* L.	Tertiary
Fabaceae	Clover	*Trifolium pratense* L.	*Trifolium congestum* Guss.	Tertiary
Fabaceae	Clover	*Trifolium pratense* L.	*Trifolium dasyurum* C.Presl	Tertiary
Fabaceae	Clover	*Trifolium pratense* L.	*Trifolium dichroanthum* Boiss.	Tertiary
Fabaceae	Clover	*Trifolium pratense* L.	*Trifolium echinatum* M.Bieb.	Tertiary
Fabaceae	Clover	*Trifolium pratense* L.	*Trifolium gemellum* Pourr. ex Willd.	Tertiary
Fabaceae	Clover	*Trifolium repens* L.	*Trifolium glomeratum* L.	Tertiary
Fabaceae	Clover	*Trifolium pratense* L.	*Trifolium hirtum* All.	Tertiary
Fabaceae	Clover	*Trifolium repens* L.	*Trifolium isthmocarpum* Brot.	Tertiary
Fabaceae	Clover	*Trifolium pratense* L.	*Trifolium juliani* Batt.	Tertiary
Fabaceae	Clover	*Trifolium pratense* L.	*Trifolium lappaceum* L.	Tertiary
Fabaceae	Clover	*Trifolium pratense* L.	*Trifolium leucanthum* M.Bieb.	Tertiary
Fabaceae	Clover	*Trifolium pratense* L.	*Trifolium ligusticum* Balb. ex Loisel.	Tertiary
Fabaceae	Clover	*Trifolium pratense* L.	*Trifolium medium* L.	Tertiary
Fabaceae	Clover	*Trifolium pratense* L.	*Trifolium miegeanum* Maire	Tertiary
Fabaceae	Clover	*Trifolium repens* L.	*Trifolium nigrescens* Viv.	Secondary
Fabaceae	Clover	*Trifolium repens* L.	*Trifolium nigrescens* Viv. subsp. *nigrescens*	Secondary
Fabaceae	Clover	*Trifolium pratense* L.	*Trifolium obscurum* Savi	Tertiary
Fabaceae	Clover	*Trifolium pratense* L.	*Trifolium ochroleucon* Huds.	Tertiary
Fabaceae	Clover	*Trifolium pratense* L.	*Trifolium pallidum* Waldst. et Kit.	Tertiary
Fabaceae	Clover	*Trifolium pratense* L.	*Trifolium phleoides* Pourr. ex Willd.	Tertiary
Fabaceae	Clover	*Trifolium pratense* L.	*Trifolium pratense* L.	Primary
Fabaceae	Clover	*Trifolium pratense* L.	*Trifolium purpureum* Loisel.	Tertiary
Fabaceae	Clover	*Trifolium pratense* L.	*Trifolium purpureum* Loisel. var. *purpureum*	Tertiary
Fabaceae	Clover	*Trifolium repens* L.	*Trifolium repens* L.	Primary
Fabaceae	Clover	*Trifolium repens* L.	*Trifolium repens* L. var. *giganteum* Lagr. Foss.	Primary
Fabaceae	Clover	*Trifolium pratense* L.	*Trifolium retusum* L.	Tertiary
Fabaceae	Clover	*Trifolium pratense* L.	*Trifolium scabrum* L.	Tertiary
Fabaceae	Clover	*Trifolium alexandrinum* L.	*Trifolium scutatum* Boiss.	Tertiary
Fabaceae	Clover	*Trifolium pratense* L.	*Trifolium squarrosum* L.	Tertiary
Fabaceae	Clover	*Trifolium pratense* L.	*Trifolium stellatum* L.	Tertiary
Fabaceae	Clover	*Trifolium pratense* L.	*Trifolium stellatum* L. var. *stellatum*	Tertiary

Family	Crop	Crop Latin Name	CWR	Genepool
Fabaceae	Clover	*Trifolium pratense* L.	*Trifolium striatum* L.	Tertiary
Fabaceae	Clover	*Trifolium subterraneum* L.	*Trifolium subterraneum* L. subsp. *brachycalycinum* Katzn. & F.H.W.Morley	Primary
Fabaceae	Clover	*Trifolium subterraneum* L.	*Trifolium subterraneum* L.	Primary
Fabaceae	Clover	*Trifolium subterraneum* L.	*Trifolium subterraneum* L. subsp. *subterraneum*	Primary
Fabaceae	Clover	*Trifolium repens* L.	*Trifolium suffocatum* L.	Tertiary
Fabaceae	Clover	*Trifolium repens* L.	*Trifolium thalii* Vill.	Tertiary
Fabaceae	Clover	*Trifolium repens* L.	*Trifolium uniflorum* L.	Tertiary
Poaceae	Wheat	*Triticum aestivum* L.	*Triticum monococcum* L.	Primary
Poaceae	Wheat	*Triticum aestivum* L.	*Triticum timopheevii* (Zhuk.) Zhuk.	Primary
Poaceae	Wheat	*Triticum aestivum* L.	*Triticum turgidum* Steud.	Primary
Poaceae	Wheat	*Triticum aestivum* L.	*Triticum urartu* Thumanjan ex Gandilyan	Primary
Fabaceae	Garden pea	Pisum sativum L.	*Vavilovia formosa* (Steven) Fed.	Tertiary
Fabaceae	Vetch	*Vicia ervilia* (L.) Willd.	*Vicia articulata* Hornem.	Secondary
Fabaceae	Narbon vetch	*Vicia narbonensis* L.	*Vicia bithynica* (L.) L.	Secondary
Fabaceae	Narbon vetch	*Vicia narbonensis* L.	*Vicia eristalioides* Maxted	Secondary
Fabaceae	Vetch	*Vicia ervilia* (L.) Willd.	*Vicia ervilia* (L.) Willd.	Primary
Fabaceae	Vetch	*Vicia sativa* L.	*Vicia grandiflora* Scop.	Tertiary
Fabaceae	Vetch	*Vicia pannonica* Crantz	*Vicia hybrida* L.	Secondary
Fabaceae	Narbon vetch	*Vicia narbonensis* L.	*Vicia hyaeniscyamus Mout.*	Secondary
Fabaceae	Vetch	*Vicia narbonensis* L.	*Vicia johannis* Tamamsch	Secondary
Fabaceae	Vetch	*Vicia narbonensis* L.	*Vicia kalakhensis* Khattab, Maxted and Bisby	Secondary
Fabaceae	Vetch	*Vicia sativa* L.	*Vicia lathyroides* L.	Secondary
Fabaceae	Vetch	*Vicia pannonica* Crantz	*Vicia lutea* L.	Secondary
Fabaceae	Vetch	*Vicia sativa* L.	*Vicia melanops* Sm.	Tertiary
Fabaceae	Faba Bean	*Vicia faba* L.	*Vicia narbonensis* L.	Tertiary
Fabaceae	Narbon bean	*Vicia narbonensis* L.	*Vicia narbonensis* L. var. *salmonea* (Mout.) H.Schafer	Primary
Fabaceae	Narbon bean	*Vicia narbonensis* L.	*Vicia narbonensis* L. var. *aegyptiaca* Kornhuber ex Asch. et Schweinf.	Primary
Fabaceae	Narbon bean	*Vicia narbonensis* L.	*Vicia narbonensis* L. var. *affinis* Kornhuber ex Asch. et Schweinf.	Primary
Fabaceae	Narbon bean	*Vicia narbonensis* L.	*Vicia narbonensis* L. var. *narbonensis*	Primary
Fabaceae	Vetch	*Vicia sativa* L.	*Vicia sativa* L. subsp. *amphicarpa* (L.) Batt.	Primary
Fabaceae	Vetch	*Vicia sativa* L.	*Vicia sativa* L. subsp. *nigra* (L.) Ehrh.	Primary
Fabaceae	Faba Bean	*Vicia narbonensis* L.	*Vicia serratifolia* Jacq.	Secondary
Vitaceae	Grapes	*Vitis vinifera* L.	*Vitis vinifera* L.	Primary

**Family**	**Crop**	**Crop Latin Name**	**CWR**	**Genepool**
Vitaceae	Grapes	*Vitis vinifera* L.	*Vitis vinifera* L. subsp. *sylvestris* Hegi	Primary

Supplementary Table S2. Red List status of the human and animal food wild relatives that occur in any of the countries in the WANA region and that have been assessed (data analysed from: https://www.iucnredlist.org/). [Critically Endangered (CR), Endangered (EN), Vulnerable (VU), Near Threatened (NT), Least Concern (LC), Data Deficient (DD)]

Taxon	Population Trend	RL Category	RL Criteria	Level of Assessment	Year of Assessment
*Aegilops bicornis**	Unknown	NT	B2ab(iii,v)	Global	2017
*Aegilops biuncialis**	Stable	LC	-	Global	2017
Aegilops caudata	Stable	LC	-	Global	2017
*Aegilops columnaris**	Stable	LC	-	Global	2017
*Aegilops comosa**	Stable	LC	-	Global	2017
*Aegilops crassa**	Unknown	LC	-	Global	2017
*Aegilops cylindrica**	Unknown	LC	-	Global	2017
*Aegilops geniculata**	Stable	LC	-	Global	2017
Aegilops juvenalis	Unknown	DD	-	Global	2017
*Aegilops kotschyi**	Unknown	LC	-	Global	2017
*Aegilops longissima**	Decreasing	LC	-	Global	2017
*Aegilops neglecta**	Stable	LC	-	Global	2017
*Aegilops peregrina**	Unknown	LC	-	Global	2017
Aegilops searsii	Stable	LC	-	Global	2017
*Aegilops sharonensis**	Decreasing	VU	A3c	Global and Mediterranean	2017
*Aegilops speltoides**	Stable	LC	-	Global	2017
*Aegilops tauschii**	Stable	LC	-	Global	2017
*Aegilops triuncialis**	Increasing	LC	-	Global	2017
*Aegilops umbellulata**	Stable	LC	-	Global	2017
Aegilops vavilovii	Unknown	LC	-	Global	2017
*Aegilops ventricosa**	Unknown	LC	-	Global	2017
Agrostis canina	Stable	LC	-	Global	2014
Agrostis lachnantha	Unknown	LC	-	Global	2015
Agrostis nevadensis	Decreasing	LC	-	Global and Mediterranean	2018
Agrostis olympica	Stable	LC	-	Global	2013
Agrostis stolonifera	Stable	LC	-	Global	2014
Agrostis tenerrima	Decreasing	NT	-	Global and Mediterranean	2010
Allium amethystinum	Unknown	LC	-	Global and Mediterranean	2017
*Allium ampeloprasum**	Unknown	LC	-	Global	2016
Allium asarense	Unknown	DD	-	Global	2016
*Allium atroviolaceum**	Unknown	DD	-	Global	2016

Taxon	Population Trend	RL Category	RL Criteria	Level of Assessment	Year of Assessment
Allium autumnale	Unknown	LC	-	Global, Europe and Mediterranean	2011
Allium baeticum	Unknown	LC	-	Global and Mediterranean	2019
Allium basalticum	Decreasing	EN	B2ab(i,ii,iii,v)	Global and Mediterranean	2017
Allium baytopiorum	Stable	CR	B2ab(iii)	Global	2014
Allium birkinshawii	Unknown	DD	-	Global	2018
*Allium bourgeaui**	Unknown	DD	-	Global and Mediterranean	2016
Allium callimischon	Unknown	LC	-	Global and Mediterranean	2017
Allium calocephalum	Decreasing	NT	B1ab(iii)	Global	2018
Allium carmeli	Stable	NT	-	Global and Mediterranean	2017
Allium chamaemoly	Unknown	LC	-	Global and Mediterranean	2018
Allium colchicifolium	Stable	LC	-	Global	2013
*Allium commutatum**	Stable	LC	-	Global and Mediterranean	2018
Allium cyprium	Stable	LC	-	Global and Mediterranean	2017
Allium czelghauricum	Stable	CR	B2ab(iii)	Global	2014
Allium drusorum	Unknown	DD	-	Global and Mediterranean	2017
Allium duriaeanum	Unknown	EN	D	Global and Mediterranean	2019
Allium exaltatum	Decreasing	VU	D1+2	Global, Europe and Mediterranean	2011
Allium feinbergii	Stable	LC	-	Global and Mediterranean	2017
Allium galileum	Stable	LC	-	Global and Mediterranean	2017
Allium hermoneum	Stable	LC	-	Global and Mediterranean	2017
Allium koenigianum	Unknown	DD	-	Global	2014
Allium libani	Stable	NT	-	Global and Mediterranean	2017
Allium makmelianum	Unknown	NT	-	Global and Mediterranean	2017
Allium marathasicum	Stable	CR	B1ab(iii)+2ab(iii)	Global and Mediterranean	2017
Allium meikleanum	Decreasing	NT	B1a(i)b(iii)+2a(i)b(iii)	Global and Mediterranean	2017
Allium meronense	Decreasing	EN	B1ab(ii,iii,v)+2ab(ii,iii,v)	Global and Mediterranean	2017
Allium multiflorum	Unknown	LC	-	Global and Mediterranean	2018
Allium noeanum	Unknown	EN	B2ab(ii,iii,v)	Global	2019
Allium notabile	Unknown	DD	-	Global	2017
Allium oschaninii	Unknown	DD	-	Global	2016
Allium peroninianum	Decreasing	EN	B1ab(iii)+2ab(iii)	Global	2017
Allium polyanthum	Stable	LC	-	Global	2019
Allium pseudoalbidum	Stable	EN	B2ab(iii)	Global	2014

Taxon	Population Trend	RL Category	RL Criteria	Level of Assessment	Year of Assessment
Allium pseudocalyptratum	Decreasing	EN	B1ab(iii,v)+2ab(iii,v)	Global and Mediterranean	2020
Allium pseudophanerantherum	Stable	VU	D2	Global and Mediterranean	2017
Allium pseudostamineum	Stable	LC	-	Global and Mediterranean	2017
Allium pustulosum	Unknown	DD	-	Global	2019
Allium rubrovittatum	Stable	LC	-	Global, Europe and Mediterranean	2018
Allium sannineum	Decreasing	EN	B1ab(i,ii,iii,iv,v)+2ab(i,ii,iii,iv,v)	Global and Mediterranean	2020
Allium scaberrimum	Decreasing	VU	B2ab(ii,iii,iv,v)	Global	2018
*Allium scabriscapum**	Unknown	DD	-	Global	2016
*Allium schoenoprasum**	Stable	LC	-	Global	2016
Allium scorzonerifolium	Unknown	LC	-	Global and Mediterranean	2018
Allium sieheanum	Decreasing	LC	-	Global	2018
Allium sipyleum	Unknown	LC	-	Global and Mediterranean	2017
Allium sprengeri	Unknown	DD	-	Global and Mediterranean	2017
Allium therinanthum	Decreasing	EN	B1ab(ii,iii,iv,v)+2ab(ii,iii,iv,v)	Global and Mediterranean	2017
Allium trachycoleum	Unknown	DD	-	Global	2019
Allium trichocnemis	Unknown	VU	B1ab(iii)+2ab(iii); D1	Global and Mediterranean	2019
Allium triquetrum	Stable	LC	-	Global and Mediterranean	2018
Allium urmiense	Unknown	DD	-	Global	2018
Allium willeanum	Stable	LC	-	Global, Europe and Mediterranean	2011
Alopecurus aequalis	Unknown	LC	-	Global	2014
Alopecurus arundinaceus	Unknown	LC	-	Global	2014
Alopecurus creticus	Unknown	LC	-	Global and Mediterranean	2014
Alopecurus himalaicus	Stable	LC	-	Global	2013
Alopecurus setarioides	Unknown	LC	-	Global and Mediterranean	2014
Amblyopyrum muticum	Decreasing	EN	B2ab(iii)	Global	2017
Apium crassipes	Decreasing	NT	-	Global and Mediterranean	2010
Apium graveolens	Increasing	LC	-	Global	2013
Apium nodiflorum	Stable	LC	-	Global	2013
Arrhenatherum palaestinum	Unknown	LC	-	Global and Mediterranean	2017
*Asparagus acutifolius**	Unknown	LC	-	Global	2016
*Asparagus albus**	Stable	LC	-	Global and Mediterranean	2018
*Asparagus aphyllus**	Stable	LC	-	Global	2016

Taxon	Population Trend	RL Category	RL Criteria	Level of Assessment	Year of Assessment
*Asparagus maritimus**	Unknown	DD	-	Global	2016
*Asparagus officinalis**	Stable	LC	-	Global	2016
Asparagus sp. nov. A	Unknown	DD	-	Global	2004
*Asparagus tenuifolius**	Unknown	LC	-	Global	2016
*Asparagus verticillatus**	Stable	LC	-	Global	2016
Astragalus abnormalis	Unknown	DD	-	Global	2012
Astragalus acetabulosus	Unknown	EN	B1ab(iii)	Global	2012
Astragalus acmophylloides	Decreasing	CR	B2ab(i,ii,iii)	Global	2014
Astragalus alamliensis	Unknown	EN	B1ab(iii)	Global	2012
Astragalus albispinus	Stable	LC	-	Global	2012
Astragalus angulosus	Decreasing	VU	B1ab(iii)+2ab(iii)	Global and Mediterranean	2020
Astragalus atricapillus	Stable	NT	-	Global	2012
Astragalus aucheri	Stable	LC	-	Global	2012
Astragalus bourgaeanus	Stable	LC	-	Global	2012
Astragalus cedreti	Decreasing	EN	B1ab(iii)+2ab(iii)	Global and Mediterranean	2020
Astragalus cephalotes	Stable	LC	-	Global	2012
Astragalus coarctatus	Unknown	DD	-	Global	2014
Astragalus commixtus	Stable	LC	-	Global	2012
Astragalus confusus	Stable	LC	-	Global	2012
Astragalus crenatus	Stable	LC	-	Global	2012
Astragalus denudatus	Stable	LC	-	Global	2012
Astragalus ehdenensis	Decreasing	EN	B1ab(iii)+2ab(iii)	Global and Mediterranean	2020
Astragalus eliasianus	Decreasing	CR	B1ab(i,ii,iii,v)+2ab(i,ii,iii,v); C2a(i,ii); D	Global	2014
Astragalus floccosus	Stable	LC	-	Global	2012
Astragalus froedinii	Stable	LC	-	Global	2012
Astragalus hirsutissimus	Decreasing	EN	B1ab(iii)+2ab(iii)	Global and Mediterranean	2020
Astragalus imbricatus	Unknown	DD	-	Global	2014
Astragalus kahiricus	Stable	LC	-	Global	2012
Astragalus kurnet-es-saudae	Decreasing	CR	B1ab(iii)	Global and Mediterranean	2020
Astragalus lanatus	Decreasing	EN	B1ab(iii)+2ab(iii)	Global and Mediterranean	2020
Astragalus leucophanus	Unknown	DD	-	Global	2012
Astragalus longivexillatus	Unknown	CR	B2ab(i,ii,iii)	Global	2014

Taxon	Population Trend	RL Category	RL Criteria	Level of Assessment	Year of Assessment
Astragalus myriacanthus	Stable	LC	-	Global	2012
Astragalus nigrocalycinus	Decreasing	CR	B1ab(i,ii,iii)+2ab(i,ii,iii)	Global	2014
Astragalus olurensis	Decreasing	CR	B1ab(i,ii,iii)+2ab(i,ii,iii)	Global	2014
Astragalus orthocarpoides	Unknown	DD	-	Global	2012
Astragalus paralurges	Unknown	DD	-	Global	2012
Astragalus plebejus	Unknown	DD	-	Global	2012
Astragalus ruscifolius	Unknown	NT	-	Global	2012
Astragalus sesameus	Stable	LC	-	Global	2012
Astragalus sonamerensis	Unknown	DD	-	Global	2014
Astragalus stevenianus	Stable	LC	-	Global	2012
Astragalus subalpinus	Unknown	DD	-	Global	2012
Astragalus submaculatus	Stable	LC	-	Global	2012
Astragalus tatlii	Unknown	CR	B1ab(i,ii,iii)+2ab(i,ii,iii)	Global	2014
Astragalus trabzonicus	Decreasing	CR	B1ab(i,ii,iii)+2ab(i,ii,iii)	Global	2014
Astragalus transnominatus	Decreasing	EN	B1ab(iii)+2ab(iii)	Global and Mediterranean	2020
Astragalus tribuloides	Stable	LC	-	Global	2012
Astragalus voronovianus	Unknown	DD	-	Global	2014
Atriplex farinosa	Unknown	LC	-	Global	2022
Avena abyssinica	Unknown	LC	-	Global	2016
*Avena fatua**	Stable	LC	-	Global	2016
*Avena hybrida**	Unknown	DD	-	Global	2016
*Avena murphyi**	Decreasing	EN	B2ab(ii,iii)	Global and Mediterranean	2018
*Avena sterilis**	Stable	LC	-	Global	2016
Barbarea integrifolia	Unknown	LC	-	Global	2014
Barbarea lutea	Decreasing	EN	B1ab(i,ii,iii)+2ab(i,ii,iii)	Global	2014
Barbarea macrocarpa	Decreasing	EN	B1ab(iii)+2ab(iii)	Global and Mediterranean	2020
Barbarea plantaginea	Unknown	LC	-	Global	2014
Barbarea vulgaris	Stable	LC	-	Global	2014
*Brassica barrelieri**	Unknown	LC	-	Global	2020
Brassica cretica	Increasing	LC	-	Global	2020
*Brassica elongata**	Unknown	LC	-	Global	2020
*Brassica fruticulosa**	Unknown	LC	-	Global	2020
Brassica hilarionis	Decreasing	EN	B1ab(iii,v)+2ab(iii,v); C2a(i)	Global and Europe	2011

Taxon	Population Trend	RL Category	RL Criteria	Level of Assessment	Year of Assessment
Carex acuta	Stable	LC	-	Global	2014
Carex acutiformis	Stable	LC	-	Global and Pan-Africa	2017
Carex appropinquata	Stable	LC	-	Global	2014
Carex atherodes	Unknown	LC	-	Global	2014
Carex caespitosa	Unknown	LC	-	Global	2014
Carex canescens	Stable	LC	-	Global	2013
Carex cilicica	Unknown	LC	-	Global	2014
Carex cyprica	Unknown	DD	-	Global and Mediterranean	2017
Carex davalliana	Unknown	LC	-	Global	2014
Carex diandra	Unknown	LC	-	Global	2014
Carex diluta	Unknown	LC	-	Global	2014
Carex distachya	Stable	LC	-	Global	2013
Carex distans	Stable	LC	-	Global	2013
Carex divisa	Stable	LC	-	Global	2014
Carex echinata	Stable	LC	-	Global	2016
*Carex elata**	Stable	LC	-	Global	2014
Carex extensa	Stable	LC	-	Global	2014
Carex fissirostris	Unknown	EN	B1ab(iii,v)	Global, Mediterranean and Pan-Africa	2010
Carex flava	Unknown	LC	-	Global	2014
Carex heleonastes	Unknown	DD	-	Global	2016
Carex helodes	Decreasing	NT	B2b(iii)	Global and Mediterranean	2018
Carex hispida	Stable	LC	-	Global and Mediterranean	2010
Carex iraqensis	Unknown	NT	-	Global	2014
Carex limosa	Stable	LC	-	Global	2014
Carex magellanica	Stable	LC	-	Global	2014
Carex melanorrhyncha	Unknown	DD	-	Global	2014
Carex microglochin	Stable	LC	-	Global	2014
Carex nigra	Stable	LC	-	Global	2014
Carex oederi	Unknown	LC	-	Global	2016
Carex oedipostyla	Stable	LC	-	Global and Mediterranean	2018
Carex olbiensis	Unknown	LC	-	Global and Mediterranean	2018
Carex orbicularis	Unknown	LC	-	Global	2014
Carex otrubae	Unknown	LC	-	Global	2014

Taxon	Population Trend	RL Category	RL Criteria	Level of Assessment	Year of Assessment
*Carex paniculata**	Stable	LC	-	Global	2014
*Carex pseudocyperus**	Stable	LC	-	Global	2014
Carex pseudofoetida	Unknown	LC	-	Global	2014
Carex punctata	Unknown	LC	-	Global	2014
Carex remota	Stable	LC	-	Global	2013
*Carex riparia**	Stable	LC	-	Global	2014
Carex rostrata	Stable	LC	-	Global	2014
Carex songorica	Unknown	LC	-	Global	2014
Carex stenophylla	Unknown	LC	-	Global	2013
Carex troodi	Unknown	NT	-	Global, Europe and Mediterranean	2011
Carex umbrosa	Unknown	LC	-	Global	2014
Carex vesicaria	Stable	LC	-	Global	2014
Carex vulpina	Stable	LC	-	Global	2013
Carex vulpinoidea	Unknown	LC	-	Global	2016
Carum asinorum	Decreasing	EN	B2ab(iii,v)	Global, Mediterranean and Pan-Africa	2010
Carum foetidum	Unknown	NT	-	Global and Mediterranean	2010
Carum jahandiezii	Unknown	NT	-	Global, Mediterranean and Pan-Africa	2010
*Carum lacuum**	Unknown	VU	B2ab(iii)	Global, Mediterranean and Pan-Africa	2010
*Castanea sativa**	Stable	LC	-	Global	2018
Cenchrus abyssinicus	Unknown	LC	-	Global	2017
Cenchrus ciliaris	Stable	LC	-	Global	2017
Cenchrus flaccidus	Stable	LC	-	Global	2018
Cenchrus geniculatus	Stable	LC	-	Global	2017
Cenchrus macrourus	Stable	LC	-	Global	2018
Cenchrus orientalis	Stable	LC	-	Global	2017
Cenchrus pennisetiformis	Unknown	LC	-	Global	2018
Cenchrus purpureus	Stable	LC	-	Global	2017
Cenchrus setaceus	Unknown	LC	-	Global	2018
Cenchrus setiger	Stable	LC	-	Global	2018
Cenchrus sieberianus	Stable	LC	-	Global	2018
Cenchrus stramineus	Unknown	LC	-	Global	2018
Cenchrus unisetus	Unknown	LC	-	Global	2018
Cenchrus violaceus	Unknown	LC	-	Global	2018

Taxon	Population Trend	RL Category	RL Criteria	Level of Assessment	Year of Assessment
*Ceratonia siliqua**	Decreasing	LC	-	Global	2017
Chenopodium fasciculosum	Unknown	LC	-	Global	2022
*Cicer bijugum**	Unknown	EN	B2ab(iii)	Global	2016
*Cicer echinospermum**	Stable	LC	-	Global	2012
Cicer oxyodon	Stable	LC	-	Global	2012
*Cicer pinnatifidum**	Unknown	DD	-	Global	2016
*Cicer reticulatum**	Decreasing	NT	B2b(iii)	Global	2016
Citrus medica	Decreasing	LC	-	Global	2021
Coronilla valentina	Stable	LC	-	Global	2012
*Corylus avellana**	Stable	LC	-	Global	2014
Corylus colurna	Unknown	LC	-	Global	2014
Corylus maxima	Unknown	DD	-	Global and Europe	2018
Crataegus azarolus	Unknown	LC	-	Global	2017
Crataegus laciniata	Unknown	LC	-	Global	2018
Crataegus meyeri	Unknown	DD	-	Global	2018
Crataegus monogyna	Unknown	LC	-	Global	2017
Crataegus pentagyna	Unknown	LC	-	Global	2018
Crataegus rhipidophylla	Unknown	LC	-	Global	2018
Crataegus songarica	Stable	LC	-	Global	2019
Crataegus turcicus	Decreasing	CR	B2ab(ii,iii); D	Global	2014
Crataegus wattiana	Unknown	LC	-	Global	2021
Crocus aerius	Stable	EN	B1ab(iii)+2ab(iii)	Global	2014
Crocus boissieri	Unknown	DD	-	Global and Mediterranean	2014
Crocus cyprius	Unknown	VU	D2	Global, Europe and Mediterranean	2011
Crocus hartmannianus	Unknown	VU	D2	Global, Europe and Mediterranean	2011
Crocus hyemalis	Stable	LC	-	Global and Mediterranean	2017
Crocus nevadensis	Unknown	LC	-	Global	2018
Crocus ochroleucus	Stable	LC	-	Global and Mediterranean	2017
Crocus veneris	Stable	LC	-	Global and Mediterranean	2017
Cydonia oblonga	Unknown	LC	-	Global	2021
Cyperus alopecuroides	Stable	LC	-	Global	2018
Cyperus alternifolius	Increasing	LC	-	Global	2018
Cyperus alulatus	Unknown	LC	-	Global	2013

Taxon	Population Trend	RL Category	RL Criteria	Level of Assessment	Year of Assessment
Cyperus arenarius	Stable	LC	-	Global	2011
Cyperus articulatus	Stable	LC	-	Global	2018
Cyperus compressus	Increasing	LC	-	Global	2018
Cyperus cyprius	Unknown	VU	D2	Global, Europe and Mediterranean	2011
Cyperus difformis	Stable	LC	-	Global	2018
Cyperus digitatus	Stable	LC	-	Global	2020
Cyperus dilatatus	Unknown	LC	-	Global	2013
Cyperus dives	Increasing	LC	-	Global	2018
Cyperus dubius	Stable	LC	-	Global	2013
Cyperus elegantulus	Stable	LC	-	Global	2018
Cyperus esculentus	Unknown	LC	-	Global	2013
Cyperus flavescens	Stable	LC	-	Global	2018
Cyperus fuscus	Unknown	LC	-	Global	2013
Cyperus glaber	Stable	LC	-	Global	2014
Cyperus glomeratus	Stable	LC	-	Global	2014
Cyperus hamulosus	Unknown	LC	-	Global	2014
Cyperus haspan	Stable	LC	-	Global	2018
Cyperus iria	Unknown	LC	-	Global	2020
Cyperus laevigatus	Stable	LC	-	Global	2018
Cyperus latifolius	Stable	LC	-	Global	2018
Cyperus longus	Stable	LC	-	Global	2020
Cyperus maculatus	Stable	LC	-	Global	2020
Cyperus michelianus	Stable	LC	-	Global	2020
Cyperus microbolbos	Unknown	DD	-	Global and Pan-Africa	2010
Cyperus mundii	Stable	LC	-	Global	2018
Cyperus nitidus	Stable	LC	-	Global	2018
Cyperus nutans	Stable	LC	-	Global	2013
Cyperus odoratus	Stable	LC	-	Global	2021
Cyperus papyrus	Stable	LC	-	Global	2018
Cyperus plateilema	Unknown	LC	-	Global and Pan-Africa	2017
Cyperus polystachyos	Stable	LC	-	Global	2018
Cyperus pumilus	Stable	LC	-	Global	2018
Cyperus rigidifolius	Stable	LC	-	Global	2013

Taxon	Population Trend	RL Category	RL Criteria	Level of Assessment	Year of Assessment
Cyperus rotundus	Stable	LC	-	Global	2018
Cyperus sanguinolentus	Stable	LC	-	Global	2018
Cyperus schimperianus	Unknown	LC	-	Global	2017
Cyperus sesquiflorus	Stable	LC	-	Global	2018
Cyperus squarrosus	Stable	LC	-	Global	2018
Cyperus tenuiculmis	Stable	LC	-	Global	2018
Cyperus wissmannii	Unknown	DD	-	Global	2013
Cytisus arboreus	Stable	LC	-	Global	2018
Cytisus pulvinatus	Unknown	DD	-	Global	2012
Cytisus striatus	Increasing	LC	-	Global	2012
Cytisus syriacus	Decreasing	EN	B1ab(iii)+2ab(iii)	Global and Mediterranean	2020
*Daucus aureus**	Stable	LC	-	Global	2018
*Daucus carota**	Stable	LC	-	Global	2021
*Daucus crinitus**	Unknown	LC	-	Global	2018
Daucus della-cellae	Unknown	LC	-	Global	2018
Daucus durieua	Stable	LC	-	Global	2018
*Daucus gracilis**	Unknown	LC	-	Global	2018
Daucus involucratus	Stable	LC	-	Global	2018
*Daucus jordanicus**	Stable	LC	-	Global	2018
Daucus littoralis	Stable	LC	-	Global	2018
Daucus mirabilis	Unknown	VU	D1	Global	2018
*Daucus muricatus**	Stable	LC	-	Global	2018
Daucus pumilus	Stable	LC	-	Global	2018
*Daucus sahariensis**	Unknown	LC	-	Global	2018
*Digitaria debilis**	Unknown	LC	-	Global	2020
Dioscorea lanata	Unknown	LC	-	Global	2004
Dioscorea orientalis	Stable	LC	-	Global and Mediterranean	2017
Diospyros lotus	Stable	LC	-	Global	2023
Diospyros mespiliformis	Unknown	LC	-	Global	2021
Drimia fugax	Stable	LC	-	Global and Mediterranean	2018
Drimia maritima	Unknown	LC	-	Global and Mediterranean	2018
Drimia porphyrostachys	Unknown	DD	-	Global	2004
Drimia undata	Stable	LC	-	Global	2014

Taxon	Population Trend	RL Category	RL Criteria	Level of Assessment	Year of Assessment
Echinochloa colona	Stable	LC	-	Global	2020
Echinochloa crus-galli	Increasing	LC	-	Global	2013
Echinochloa frumentacea	Stable	LC	-	Global	2018
Echinochloa picta	Stable	LC	-	Global	2011
Echinochloa pyramidalis	Stable	LC	-	Global	2020
Echinochloa stagnina	Unknown	LC	-	Global	2020
Eleusine africana	Stable	LC	-	Global	2016
Eleusine floccifolia	Stable	LC	-	Global	2016
Eleusine indica	Increasing	LC	-	Global	2011
Eleusine multiflora	Unknown	LC	-	Global	2021
Elymus elongatus	Decreasing	LC	-	Global	2019
Elymus farctus	Unknown	LC	-	Global	2019
Elymus hispidus	Stable	LC	-	Global	2019
Eragrostis aethiopica	Stable	LC	-	Global	2013
Eragrostis atrovirens	Stable	LC	-	Global	2020
Eragrostis gangetica	Stable	LC	-	Global	2020
Eragrostis japonica	Stable	LC	-	Global	2020
Eragrostis unioloides	Increasing	LC	-	Global	2011
Festuca pontica	Decreasing	EN	B1ab(i,ii,iii,v)+2ab(i,ii,iii,v)	Global	2014
Festuca varia	Unknown	LC	-	Global	2014
Festuca xenophontis	Decreasing	EN	B1ab(i,ii,iii,v)+2ab(i,ii,iii,v)	Global	2014
Festuca yemenensis	Unknown	VU	D2	Global	2013
*Ficus carica**	Increasing	LC	-	Global	2023
Ficus exasperata	Stable	LC	-	Global	2019
Ficus glumosa	Stable	LC	-	Global	2020
Ficus ingens	Stable	LC	-	Global	2019
Ficus johannis	Unknown	LC	-	Global	2021
Ficus microcarpa	Stable	LC	-	Global	2019
Ficus palmata	Unknown	LC	-	Global	2022
Ficus populifolia	Stable	LC	-	Global	2019
Ficus sur	Stable	LC	-	Global	2020
Ficus sycomorus	Stable	LC	-	Global	2019
Ficus vasta	Stable	LC	-	Global	2019

Taxon	Population Trend	RL Category	RL Criteria	Level of Assessment	Year of Assessment
Hedysarum flexuosum	Decreasing	NT	-	Global	2012
Hedysarum formosum	Decreasing	LC	-	Global	2021
Hippophae rhamnoides	Stable	LC	-	Global	2018
Hordeum bogdanii	Stable	LC	-	Global	2016
*Hordeum brevisubulatum**	Stable	LC	-	Global	2016
*Hordeum bulbosum**	Stable	LC	-	Global	2016
*Hordeum marinum**	Stable	LC	-	Global	2016
*Hordeum murinum**	Stable	LC	-	Global	2016
*Hordeum secalinum**	Decreasing	LC	-	Global	2016
Ilex aquifolium	Stable	LC	-	Global	2018
Ipomoea aquatica	Unknown	LC	-	Global	2018
Ipomoea cairica	Stable	LC	-	Global	2017
Ipomoea pes-caprae	Stable	LC	-	Global	2021
Ipomoea sagittata	Stable	LC	-	Global	2021
Ipomoea triloba	Stable	LC	-	Global	2019
Isatis arenaria	Unknown	DD	-	Global and Europe	2011
Isatis glastifolia	Unknown	VU	B1ab(iii)+2ab(iii)	Global	2014
Isatis ornithorhynchus	Unknown	DD	-	Global	2014
*Juglans regia**	Unknown	LC	-	Global	2017
Kosteletzkya pentacarpos	Decreasing	LC	-	Global	2014
Lactuca aurea	Stable	LC	-	Global and Europe	2011
Lactuca cyprica	Stable	NT	-	Global and Europe	2011
Lactuca tetrantha	Decreasing	VU	D2	Global and Europe	2011
*Lathyrus amphicarpos**	Decreasing	NT	B2b(ii,iii)	Global	2019
Lathyrus angulatus	Stable	LC	-	Global	2019
*Lathyrus annuus**	Stable	LC	-	Global	2019
Lathyrus aphaca	Unknown	LC	-	Global	2019
Lathyrus armenus	Unknown	EN	B2ab(iii)	Global	2019
Lathyrus aureus	Stable	LC	-	Global	2019
Lathyrus belinensis	Decreasing	CR	A3c; B1ab(i,ii,iii,v)+2ab(i,ii,iii,v)	Global and Mediterranean	2019
Lathyrus blepharicarpus	Stable	LC	-	Global	2019
Lathyrus boissieri	Unknown	NT	B2b(iii)	Global	2019
Lathyrus brachypterus	Unknown	NT	B2b(iii)	Global	2019

Taxon	Population Trend	RL Category	RL Criteria	Level of Assessment	Year of Assessment
Lathyrus cassius	Decreasing	NT	-	Global	2019
Lathyrus chloranthus	Stable	LC	-	Global	2019
Lathyrus chrysanthus	Unknown	NT	B2b(iii,v)	Global	2019
*Lathyrus cicera**	Stable	LC	-	Global	2019
Lathyrus cilicicus	Decreasing	EN	B2ab(iii,v)	Global	2019
Lathyrus ciliolatus	Unknown	LC	-	Global	2019
*Lathyrus clymenum**	Unknown	LC	-	Global	2019
Lathyrus cyaneus	Unknown	VU	D1	Global	2019
Lathyrus czeczottianus	Unknown	NT	B2b(iii)	Global	2019
Lathyrus digitatus	Unknown	LC	-	Global	2019
Lathyrus elongatus	Unknown	NT	B2a	Global	2019
Lathyrus filiformis	Unknown	LC	-	Global	2019
Lathyrus gloeosperma	Unknown	CR	C2a(i)	Global	2019
*Lathyrus gorgoni**	Stable	LC	-	Global	2019
*Lathyrus hierosolymitanus**	Stable	LC	-	Global	2019
*Lathyrus hirsutus**	Stable	LC	-	Global	2019
Lathyrus hirticarpus	Decreasing	EN	B1ab(iii,v)+2ab(iii,v)	Global	2019
Lathyrus inconspicuus	Unknown	LC	-	Global	2019
Lathyrus incurvus	Stable	LC	-	Global	2019
*Lathyrus latifolius**	Stable	LC	-	Global	2019
Lathyrus laxiflorus	Unknown	LC	-	Global	2019
Lathyrus layardii	Decreasing	EN	B2ab(iii)	Global	2019
Lathyrus libani	Unknown	EN	B2ab(iii,v)	Global	2019
Lathyrus linifolius	Unknown	LC	-	Global	2019
Lathyrus lycicus	Unknown	VU	D2	Global	2020
*Lathyrus marmoratus**	Unknown	LC	-	Global	2019
Lathyrus niger	Unknown	LC	-	Global	2019
Lathyrus nissolia	Unknown	LC	-	Global	2019
Lathyrus nivalis	Unknown	VU	B2ab(iii)	Global	2019
*Lathyrus ochrus**	Stable	LC	-	Global	2019
Lathyrus pallescens	Stable	LC	-	Global	2019
Lathyrus palustris	Unknown	LC	-	Global	2019
Lathyrus phaselitanus	Unknown	CR	B2ab(iii)	Global	2019

Taxon	Population Trend	RL Category	RL Criteria	Level of Assessment	Year of Assessment
Lathyrus pratensis	Stable	LC	-	Global	2019
*Lathyrus pseudocicera**	Unknown	LC	-	Global	2019
Lathyrus pygmaeus	Unknown	DD	-	Global	2019
Lathyrus roseus	Unknown	LC	-	Global	2019
Lathyrus rotundifolius	Stable	NT	B2b(iii)	Global	2019
Lathyrus satdaghensis	Unknown	VU	D2	Global	2019
Lathyrus saxatilis	Unknown	LC	-	Global	2019
Lathyrus setifolius	Unknown	LC	-	Global	2019
Lathyrus spathulatus	Unknown	LC	-	Global	2019
Lathyrus sphaericus	Stable	LC	-	Global	2019
Lathyrus stenolobus	Unknown	EN	B2ab(iii)	Global	2019
Lathyrus stenophyllus	Stable	NT	B2b(iii,v)	Global	2019
*Lathyrus sylvestris**	Stable	LC	-	Global	2019
Lathyrus tauricola	Unknown	EN	B2ab(iii)	Global	2019
*Lathyrus tingitanus**	Stable	LC	-	Global	2019
Lathyrus trachycarpus	Unknown	EN	B2ab(iii)	Global	2019
Lathyrus tuberosus	Stable	LC	-	Global	2019
Lathyrus tukhtensis	Unknown	VU	B2ab(iii); D2	Global	2019
Lathyrus undulatus	Decreasing	EN	B2ab(iii)	Global	2019
Lathyrus variabilis	Unknown	VU	B2ab(iii)	Global	2019
Lathyrus venetus	Unknown	LC	-	Global	2019
Lathyrus vernus	Unknown	LC	-	Global	2019
Lathyrus vinealis	Unknown	LC	-	Global	2019
Leersia hexandra	Stable	LC	-	Global	2019
Leersia oryzoides	Unknown	LC	-	Global	2016
Lemna aequinoctialis	Stable	LC	-	Global	2018
Lemna gibba	Stable	LC	-	Global	2017
Lemna minor	Stable	LC	-	Global	2019
Lemna trisulca	Stable	LC	-	Global	2017
Lemna turionifera	Unknown	LC	-	Global	2014
Leopoldia bicolor	Decreasing	NT	A2ac	Global and Mediterranean	2017
Leopoldia maritima	Decreasing	VU	B2ab(i,ii,iii,iv)	Global and Mediterranean	2020
Lepidium violaceum	Decreasing	VU	B2ab(iii)	Global, Mediterranean and Pan-Africa	2010

Taxon	Population Trend	RL Category	RL Criteria	Level of Assessment	Year of Assessment
Linum carnosulum	Decreasing	CR	B1ab(iii)	Global and Mediterranean	2020
Linum punctatum	Stable	LC	-	Global	2023
Lotus arabicus	Stable	LC	-	Global	2012
Lotus armeniacus	Decreasing	CR	C2a(i)	Global	2014
*Lotus benoistii**	Decreasing	CR	B2ab(ii,iii,iv,v)	Global, Mediterranean, Northern Africa and Pan-Africa	2010
Lotus edulis	Unknown	LC	-	Global	2022
Lotus eriosolen	Unknown	LC	-	Global	2012
Lotus herbaceus	Stable	LC	-	Global	2021
Lotus maroccanus	Stable	LC	-	Global	2012
Lotus mollis	Unknown	VU	D2	Global	2004
Lotus ononopsis	Unknown	LC	-	Global	2004
Lotus palustris	Unknown	LC	-	Global	2014
Lotus schimperi	Stable	LC	-	Global	2012
Lotus tetragonolobus	Stable	LC	-	Global	2012
*Lupinus albus**	Stable	LC	-	Global	2020
*Lupinus angustifolius**	Stable	LC	-	Global	2016
*Lupinus atlanticus**	Unknown	DD	-	Global	2020
Lupinus cosentinii	Unknown	LC	-	Global	2020
*Lupinus luteus**	Stable	LC	-	Global	2016
*Lupinus micranthus**	Stable	LC	-	Global	2016
Lupinus pilosus	Unknown	DD	-	Global	2020
*Malus florentina**	Unknown	DD	-	Global	2018
Malus orientalis	Unknown	DD	-	Global	2016
*Malus sylvestris**	Unknown	DD	-	Global and Europe	2011
Malus trilobata	Unknown	NT	B2ab(ii)	Global	2018
Medicago arborea	Stable	LC	-	Global	2016
Medicago astroites	Stable	LC	-	Global	2012
Medicago biflora	Stable	LC	-	Global	2020
Medicago bonarotiana	Stable	LC	-	Global	2020
Medicago brachycarpa	Unknown	LC	-	Global	2020
Medicago constricta	Stable	LC	-	Global	2016
*Medicago doliata**	Stable	LC	-	Global	2016

Taxon	Population Trend	RL Category	RL Criteria	Level of Assessment	Year of Assessment
*Medicago italica**	Stable	LC	-	Global	2016
*Medicago littoralis**	Stable	LC	-	Global	2016
*Medicago murex**	Stable	LC	-	Global	2016
Medicago papillosa	Decreasing	NT	B2b(v)	Global	2016
*Medicago rigidula**	Stable	LC	-	Global	2016
*Medicago rugosa**	Stable	LC	-	Global	2016
*Medicago sativa**	Stable	LC	-	Global	2016
*Medicago scutellata**	Stable	LC	-	Global	2016
Medicago secundiflora	Stable	LC	-	Global	2012
*Medicago soleirolii**	Unknown	DD	-	Global	2016
*Medicago truncatula**	Stable	LC	-	Global	2016
*Medicago turbinata**	Stable	LC	-	Global	2016
Melilotus italicus	Stable	LC	-	Global	2012
Melilotus serratifolius	Unknown	DD	-	Global	2012
*Mentha aquatica**	Stable	LC	-	Global	2014
*Mentha arvensis**	Unknown	LC	-	Global	2016
Mentha cervina	Decreasing	NT	-	Global and Mediterranean	2010
Mentha gattefossei	Decreasing	VU	B2ab(ii,iii,v)	Global, Mediterranean and Pan-Africa	2020
*Mentha longifolia**	Stable	LC	-	Global	2014
Mentha pulegium	Stable	LC	-	Global	2014
*Mentha spicata**	Stable	LC	-	Global	2014
*Mentha suaveolens**	Stable	LC	-	Global	2014
Myrtus communis	Stable	LC	-	Global	2018
Onobrychis hypargyrea	Decreasing	LC	-	Global	2021
Onobrychis viciifolia	Stable	LC	-	Global and Europe	2011
Origanum cordifolium	Unknown	VU	D2	Global and Europe	2011
Origanum ehrenbergii	Decreasing	VU	B1ab(i,ii,iii,iv,v)+2ab(i,ii,iii,iv,v)	Global and Mediterranean	2015
Origanum libanoticum	Decreasing	VU	B1ab(iii)+2ab(iii)	Global and Mediterranean	2020
Ornithopus uncinatus	Unknown	DD	-	Global	2012
Panicum acuminatum	Unknown	LC	-	Global	2016
Panicum repens	Stable	LC	-	Global	2020
Panicum rigidum	Unknown	LC	-	Global	2004
Panicum socotranum	Unknown	CR	B2ab(iii)	Global	2013

Taxon	Population Trend	RL Category	RL Criteria	Level of Assessment	Year of Assessment
Papaver libanoticum	Unknown	EN	B1ab(iii)+2ab(iii)	Global and Mediterranean	2020
Phalaris arundinacea	Unknown	LC	-	Global	2014
Phalaris truncata	Stable	LC	-	Global and Mediterranean	2010
Phleum alpinum	Stable	LC	-	Global	2013
Phleum montanum	Stable	LC	-	Global	2013
Phoenix caespitosa	Unknown	LC	-	Global	2018
Phoenix reclinata	Unknown	LC	-	Global	2017
*Phoenix theophrasti**	Unknown	Lower Risk/NT	-	Global and Mediterranean	1998
Pimpinella lazica	Decreasing	VU	B1ab(ii,iii)+2ab(ii,iii)	Global	2014
Pistacia aethiopica	Unknown	Lower Risk/NT	-	Global	1998
*Pistacia atlantica**	Decreasing	NT	A3cde	Global	2018
*Pistacia eurycarpa**	Stable	LC	-	Global	2016
*Pistacia khinjuk**	Stable	LC	-	Global	2016
*Pistacia lentiscus**	Stable	LC	-	Global	2016
*Pistacia terebinthus**	Stable	LC	-	Global	2016
Pistacia vera	Decreasing	NT	A2cd	Global	2022
Poa angustifolia	Stable	LC	-	Global	2013
Poa annua	Increasing	LC	-	Global	2013
Poa dimorphantha	Stable	LC	-	Global and Mediterranean	2010
Poa pratensis	Unknown	LC	-	Global	2016
Poa pseudobulbosa	Unknown	NT	-	Global and Mediterranean	2013
Poa schimperiana	Unknown	LC	-	Global	2013
Prosopis africana	Unknown	LC	-	Global	2019
Prosopis farcta	Stable	LC	-	Global	2022
Prunus agrestis	Decreasing	EN	B1ab(iii)+2ab(iii)	Global and Mediterranean	2020
*Prunus arabica**	Unknown	NT	B2b(ii,iii)	Global	2022
*Prunus argentea**	Unknown	DD	-	Global	2016
*Prunus avium**	Stable	LC	-	Global	2017
Prunus boissieri	Decreasing	EN	B1ab(ii,iii,iv)+2ab(ii,iii,iv)	Global	2019
*Prunus cerasifera**	Unknown	DD	-	Global	2018
*Prunus cocomilia**	Unknown	LC	-	Global	2016
*Prunus discolor**	Unknown	DD	-	Global	2018
Prunus domestica	Unknown	DD	-	Global	2017

Taxon	Population Trend	RL Category	RL Criteria	Level of Assessment	Year of Assessment
*Prunus fenzliana**	Unknown	DD	-	Global	2020
Prunus incana	Unknown	DD	-	Global	2016
Prunus korshinskyi	Unknown	VU	A1a	Global	1998
Prunus laurocerasus	Stable	LC	-	Global	2018
*Prunus lusitanica**	Decreasing	LC	-	Global	2021
*Prunus mahaleb**	Unknown	LC	-	Global	2016
*Prunus microcarpa**	Unknown	NT	B2ab(ii,iii)	Global	2022
*Prunus padus**	Stable	LC	-	Global	2016
*Prunus prostrata**	Unknown	LC	-	Global	2016
*Prunus spinosa**	Stable	LC	-	Global	2016
*Prunus webbii**	Unknown	DD	-	Global	2016
Psidium guajava	Unknown	LC	-	Global	2019
Puccinellia ciliata	Stable	DD	-	Global and Mediterranean	2014
Puccinellia iberica	Unknown	LC	-	Global and Mediterranean	2018
Punica granatum	Unknown	LC	-	Global	2020
Punica protopunica	Stable	VU	B1ab(iii)	Global	2004
Pyrus anatolica	Unknown	Lower Risk/NT	-	Global	1998
Pyrus bourgaeana	Stable	LC	-	Global	2018
*Pyrus communis**	Unknown	LC	-	Global	2017
Pyrus cordata	Unknown	LC	-	Global	2018
*Pyrus elaeagrifolia**	Unknown	DD	-	Global	2018
Pyrus hakkiarica	Unknown	DD	-	Global	1998
Pyrus nivalis	Unknown	DD	-	Global	2018
Pyrus oxyprion	Unknown	Lower Risk/NT	-	Global	1998
Pyrus salicifolia	Unknown	Lower Risk/NT	-	Global	1998
Pyrus serikensis	Unknown	VU	B1+2c	Global	1998
Pyrus spinosa	Unknown	LC	-	Global	2017
Pyrus syriaca	Stable	LC	-	Global	2018
Pyrus turcomanica	Unknown	DD	-	Global	2021
Rhamnus alaternus	Stable	LC	-	Global	2018
Rhamnus alpina	Stable	LC	-	Global	2018
Rhamnus cathartica	Stable	LC	-	Global	2018
Rhamnus staddo	Stable	LC	-	Global	2020

Taxon	Population Trend	RL Category	RL Criteria	Level of Assessment	Year of Assessment
Rhamphospermum nigrum	Stable	LC	-	Global	2020
Ribes melananthum	Unknown	DD	-	Global	2021
*Rorippa amphibia**	Stable	LC	-	Global	2014
Rorippa aurea	Unknown	LC	-	Global	2014
Rorippa austriaca	Unknown	LC	-	Global	2014
*Rorippa hayanica**	Decreasing	VU	B1ab(iii)+2ab(iii)	Global, Mediterranean and Pan-Africa	2010
Rorippa lippizensis	Stable	LC	-	Global and Europe	2011
Rorippa microphylla	Stable	LC	-	Global	2014
Rorippa sylvestris	Stable	LC	-	Global	2014
*Rumex algeriensis**	Unknown	EN	B2ab(iii,v); D	Global, Mediterranean and Pan-Africa	2010
Rumex bithynicus	Decreasing	EN	B2ab(ii)	Global	2014
Rumex ginii	Unknown	NT	-	Global, Mediterranean and Pan-Africa	2010
Rumex hydrolapathum	Stable	LC	-	Global	2014
Rumex palustris	Stable	LC	-	Global	2014
*Rumex tunetanus**	Decreasing	CR	B1ab(ii,iii)+2ab(ii,iii)	Global, Mediterranean and Pan-Africa	2010
Saccharum kajkaiense	Unknown	LC	-	Global	2013
*Saccharum spontaneum**	Stable	LC	-	Global	2020
Salvia officinalis	Stable	LC	-	Global and Europe	2014
Salvia peyronii	Stable	CR	A2c; B1ab(iii)+2ab(iii)	Global and Mediterranean	2020
Salvia taraxacifolia	Decreasing	EN	B1ab(i,ii,iii,v)+2ab(i,ii,iii,v)	Global	2020
Salvia veneris	Unknown	CR	B1ab(i,iii)	Global, Europe and Mediterranean	2006
Setaria sphacelata	Unknown	LC	-	Global	2020
Sium sisarum	Unknown	LC	-	Global	2022
Solanum adoense	Stable	LC	-	Global	2021
Solanum coagulans	Stable	LC	-	Global	2021
Solanum cordatum	Stable	LC	-	Global	2021
Solanum forskalii	Stable	LC	-	Global	2021
Solanum glabratum	Stable	LC	-	Global	2021
Solanum linnaeanum	Stable	LC	-	Global	2021
Solanum schimperianum	Stable	LC	-	Global	2021
Solanum somalense	Stable	LC	-	Global	2021
Sorghum arundinaceum	Unknown	LC	-	Global	2020
Sorghum purpureosericeum	Stable	LC	-	Global	2013

Taxon	Population Trend	RL Category	RL Criteria	Level of Assessment	Year of Assessment
Sorghum virgatum	Stable	LC	-	Global	2013
*Trifolium angustifolium**	Stable	LC	-	Global	2012
Trifolium canescens	Stable	LC	-	Global	2012
Trifolium caucasicum	Stable	LC	-	Global	2012
Trifolium clusii	Stable	LC	-	Global	2012
Trifolium incarnatum	Stable	LC	-	Global and Europe	2011
*Trifolium obscurum**	Stable	LC	-	Global	2012
*Trifolium pratense**	Stable	LC	-	Global	2012
Trifolium sannineum	Decreasing	EN	B1ab(iii)+2ab(iii)	Global and Mediterranean	2020
*Trifolium scabrum**	Stable	LC	-	Global	2012
*Trifolium subterraneum**	Stable	LC	-	Global	2012
Triticum monococcum	Stable	LC	-	Global	2017
Triticum timopheevii	Stable	LC	-	Global	2017
Triticum turgidum	Unknown	LC	-	Global	2017
Triticum urartu	Unknown	DD	-	Global	2017
Vicia abbreviata	Stable	LC	-	Global	2019
Vicia aintabensis	Decreasing	DD	-	Global	2019
Vicia anatolica	Stable	LC	-	Global	2019
*Vicia articulata**	Stable	LC	-	Global	2016
Vicia assyriaca	Unknown	DD	-	Global	2016
Vicia balansae	Stable	LC	-	Global	2019
Vicia barbazitae	Unknown	NT	B2b(iii)	Global	2016
*Vicia bithynica**	Stable	LC	-	Global	2019
Vicia ciliatula	Stable	LC	-	Global	2016
Vicia cretica	Stable	LC	-	Global	2012
Vicia cuspidata	Stable	LC	-	Global	2019
*Vicia eristalioides**	Decreasing	CR	B1ab(i,ii,iii,iv,v)+2ab(i,ii,iii,iv,v); C2a(i); D	Global	2019
*Vicia ervilia**	Stable	LC	-	Global	2016
Vicia erzurumica	Unknown	CR	B2ab(ii,iii)	Global	2014
Vicia esdraelonensis	Unknown	VU	D2	Global	2016
Vicia fulgens	Decreasing	CR	B2ab(ii,iii,iv,v)	Global, Northern Africa and Pan-Africa	2008
Vicia galeata	Unknown	LC	-	Global	2016

**Taxon**	**Population Trend**	**RL Category**	**RL Criteria**	**Level of Assessment**	**Year of Assessment**
*Vicia grandiflora**	Stable	LC	-	Global	2016
*Vicia hyaeniscyamus**	Decreasing	EN	B1ab(iii,iv)+2ab(iii,iv)	Global	2016
*Vicia hybrida**	Stable	LC	-	Global	2016
Vicia hyrcanica	Unknown	DD	-	Global	2016
Vicia incisa	Decreasing	EN	B2ab(i,ii,iii,v)	Global	2019
*Vicia johannis**	Stable	LC	-	Global	2016
*Vicia kalakhensis**	Decreasing	EN	B1ab(iv,v)+2ab(iv,v)	Global	2016
*Vicia lathyroides**	Stable	LC	-	Global	2019
*Vicia lutea**	Stable	LC	-	Global	2016
*Vicia melanops**	Stable	LC	-	Global	2016
Vicia michauxii	Decreasing	DD	-	Global	2019
Vicia mollis	Unknown	LC	-	Global	2016
*Vicia narbonensis**	Stable	LC	-	Global	2016
Vicia noeana	Stable	LC	-	Global	2019
Vicia pannonica	Stable	LC	-	Global	2016
Vicia peregrina	Stable	LC	-	Global	2019
Vicia qatmensis	Unknown	NT	B1a(i)+2a(i)	Global	2016
Vicia quadrijuga	Unknown	CR	B1ab(i,ii,iii)+2ab(i,ii,iii)	Global	2014
*Vicia sativa**	Stable	LC	-	Global	2019
Vicia sepium	Stable	LC	-	Global	2019
Vicia sericocarpa	Unknown	LC	-	Global	2016
Vicia serratifolia	Unknown	LC	-	Global	2016
Vicia tenuifolia	Stable	LC	-	Global	2012
Vicia tigridis	Unknown	VU	D2	Global	2016
Vigna luteola	Stable	LC	-	Global	2019
Vigna macrorrhyncha	Stable	LC	-	Global	2019
Vigna radiata	Unknown	LC	-	Global	2019

Supplementary Table S3. Red List status of CWR species that occur in the WANA region by crop gene pool/group, showing the percentages of the species assessed as threatened (data analysed from: https://www.iucnredlist.org/). [Critically Endangered (CR), Endangered (EN), Vulnerable (VU), Near Threatened (NT), Least Concern (LC), Data Deficient (DD)]

Related crop	Genera	No. of Species Assessed	CR	EN	VU	NT	LC	DD	% Threatened
Agrostis	*Agrostis*	6	0	0	0	1	5	0	0.0
Anise	*Pimpinella*	1	0	0	1	0	0	0	100.0
Apple	*Malus*	4	0	0	0	1	0	3	0.0
Arrhenatherum	*Arrhenatherum*	1	0	0	0	0	1	0	0.0
Asparagus	*Asparagus*	8	0	0	0	0	6	2	0.0
Astragalus	*Astragalus*	44	8	7	1	2	16	10	36.4
Barley	*Hordeum*	6	0	0	0	0	6	0	0.0
Barley, oat, wheat	*Poa*	6	0	0	0	1	5	0	0.0
Berries	*Myrtus*	1	0	0	0	0	1	0	0.0
Bird's foot	*Ornithopus*	1	0	0	0	0	0	1	0.0
Brassica complex	*Barbarea, Brassica, Isatis, Lepidium, Rorippa*	21	0	3	3	0	13	2	28.6
Broad beans, horse beans, vetches	*Vicia*	41	4	3	2	2	26	4	22.0
Buckthorn	*Rhamnus*	4	0	0	0	0	4	0	0.0
Canary seed	*Phalaris*	2	0	0	0	0	2	0	0.0
Caraway seed	*Carum*	4	0	1	1	2	0	0	50.0
Carob	*Ceratonia*	1	0	0	0	0	1	0	0.0
Carrot	*Daucus*	13	0	0	1	0	12	0	7.7
Celery	*Apium, Rhamphospermum*	4	0	0	0	1	3	0	0.0
Cenchrus	*Cenchrus*	14	0	0	0	0	14	0	0.0
Chestnut	*Castanea*	1	0	0	0	0	1	0	0.0
Chia	*Salvia*	4	2	1	0	0	1	0	75.0
Chickpea	*Cicer*	5	0	1	0	1	2	1	20.0
Chufa	*Cyperus*	43	0	0	1	0	40	2	2.3
Citrus fruits	*Citrus*	1	0	0	0	0	1	0	0.0
Clover	*Trifolium*	10	0	1	0	0	9	0	10.0
Coronilla	*Coronilla*	1	0	0	0	0	1	0	0.0
Cowpea	*Vigna*	3	0	0	0	0	3	0	0.0
Cucurbits, melons, gourds	*Kosteletzkya*	1	0	0	0	0	1	0	0.0

Related crop	Genera	No. of Species Assessed	CR	EN	VU	NT	LC	DD	% Threatened
Dates	*Phoenix*	3	0	0	0	1	2	0	0.0
Fescue	*Festuca*	4	0	2	1	0	1	0	75.0
Figs	*Ficus*	11	0	0	0	0	11	0	0.0
Fonio	*Digitaria*	1	0	0	0	0	1	0	0.0
Foxtail grass	*Alopecurus*	5	0	0	0	0	5	0	0.0
Gooseberry	*Ribes*	1	0	0	0	0	0	1	0.0
Grass pea	*Lathyrus*	66	3	9	6	9	38	1	27.3
Guava	*Psidium*	1	0	0	0	0	1	0	0.0
Hawthorn	*Crataegus*	9	1	0	0	0	7	1	11.1
Hazel	*Corylus*	3	0	0	0	0	2	1	0.0
Hedysarum	*Hedysarum*	2	0	0	0	1	1	0	0.0
Knotweed	*Sium*	1	0	0	0	0	1	0	0.0
Lettuce	*Lactuca*	3	0	0	1	1	1	0	33.3
Linseed	*Linum*	2	1	0	0	0	1	0	50.0
Lotus	*Lotus*	12	2	0	1	0	9	0	25.0
Lupin	*Lupinus*	7	0	0	0	0	5	2	0.0
Medics	*Medicago*	19	0	0	0	1	17	1	0.0
Melilotus	*Melilotus*	2	0	0	0	0	1	1	0.0
Millet	*Echinochloa, Eleusine, Eragrostis, Panicum, Setaria*	20	1	0	0	0	19	0	5.0
Oat	*Avena*	5	0	1	0	0	3	1	20.0
Onion, leek, garlic, etc	*Allium, Leopoldia*	58	3	9	5	6	22	13	29.3
Oregano	*Origanum*	3	0	0	3	0	0	0	100.0
Pears	*Pyrus*	13	0	0	1	3	5	4	7.7
Peppermint	*Mentha*	8	0	0	1	1	6	0	12.5
Persimmon	*Diospyros*	2	0	0	0	0	2	0	0.0
Pigeonpea	*Cytisus*	4	0	1	0	0	2	1	25.0
Pistachio	*Pistacia*	7	0	0	0	3	4	0	0.0
Pomegranate	*Punica*	2	0	0	1	0	1	0	50.0
Poppy seed	*Papaver*	1	0	1	0	0	0	0	100.0
Prosopis	*Prosopis*	2	0	0	0	0	2	0	0.0
Quince	*Cydonia*	1	0	0	0	0	1	0	0.0
Quinoa	*Chenopodium*	1	0	0	0	0	1	0	0.0

Related crop	Genera	No. of Species Assessed	CR	EN	VU	NT	LC	DD	% Threatened
Rice	*Lemna, Puccinellia*	7	0	0	0	0	6	1	0.0
Rice grass	*Leersia*	2	0	0	0	0	2	0	0.0
Saffron	*Crocus*	8	0	1	2	0	4	1	37.5
Sainfoin	*Onobrychis*	2	0	0	0	0	2	0	0.0
Saltbush	*Atriplex*	1	0	0	0	0	1	0	0.0
Sea buckthorn	*Hippophae*	1	0	0	0	0	1	0	0.0
Solanum	*Solanum*	8	0	0	0	0	8	0	0.0
Sorghum	*Sorghum*	3	0	0	0	0	3	0	0.0
Spinach	*Rumex*	6	1	2	0	1	2	0	50.0
Squill	*Drimia*	4	0	0	0	0	3	1	0.0
Stonefruits	*Prunus*	20	0	2	1	2	8	7	15.0
Sugarcane	*Saccharum*	2	0	0	0	0	2	0	0.0
Sweet potato	*Ipomoea*	5	0	0	0	0	5	0	0.0
Timothy grass	*Phleum*	2	0	0	0	0	2	0	0.0
Walnut	*Juglans*	1	0	0	0	0	1	0	0.0
Wheat	*Aegilops, Amblyopyrum, Elymus, Triticum*	29	0	1	1	1	24	2	6.9
Yam	*Dioscorea*	2	0	0	0	0	2	0	0.0
Yerba mate	*Ilex*	1	0	0	0	0	1	0	0.0
Various cereals (what, barley, oats, rye)	*Carex*	47	0	1	0	3	40	3	2.1
**Grand Total**		**681**	**26**	**47**	**34**	**44**	**463**	**67**	**15.7**

Supplementary Table S4. Number of threatened and non-threatened priority CWR affected by different threats (data analysed from: https://www.iucnredlist.org/).

**Threats**	**No. of threatened priority CWR**	**No. of non-threatened priority CWR**	**Total**
Livestock farming & ranching	11	32	43
Agriculture (annual & perennial non-timber crops)	6	10	16
Pollution (agricultural & forestry effluents)	4	7	11
Other ecosystem modifications	4	3	7
Housing & urban areas	3	14	17
Pollution (domestic & urban waste water)	3	1	4
Roads & railroads	2	8	10
Recreational activities	2	7	9
Logging & wood harvesting	1	9	10
Habitat shifting & alteration	1	5	6
Pollution (industrial & military effluents)	1	1	2
Tourism & recreation areas	0	16	16
Fires	0	9	9
Gathering terrestrial plants	0	7	7
Invasive non-native/alien species/diseases	0	5	5
Droughts	0	5	5
Commercial & industrial areas	0	3	3
Wood & pulp plantations	0	3	3
Dams & water management/use	0	3	3
Problematic native species/diseases	0	3	3
Genetic pollution	0	3	3
Work & other activities	0	2	2
Extreme temperature	0	2	2
Other threat	0	2	2
Mining & quarrying	0	1	1
Renewable energy	0	1	1
Pollution (air-borne pollutants)	0	1	1
Avalanches/landslides	0	1	1
TOTAL	38	164	202
